# Diabetic microenvironment deteriorates the regenerative capacities of adipose mesenchymal stromal cells

**DOI:** 10.1186/s13098-024-01365-1

**Published:** 2024-06-16

**Authors:** Sara M. Ahmed, Hoda A. Elkhenany, Toka A. Ahmed, Nehal I. Ghoneim, Mohamed Abd Elkodous, Rania Hassan Mohamed, Sameh Magdeldin, Aya Osama, Ali Mostafa Anwar, Mahmoud M. Gabr, Nagwa El-Badri

**Affiliations:** 1 Center of Excellence for Stem Cells and Regenerative Medicine (CESC), Zewail City of Science and Technology, 6th of October City, Sheikh Zayed District, 6th of October City , 12582 Giza Egypt; 2 Department of surgery, Faculty of Veterinary Medicine, Alexandria University, Alexandria, Egypt; 3 Department of Biochemistry, Faculty of Science, Ain Shams University, Cairo, Egypt; 4 Proteomic and Metabolomics Research Program, Basic Research Department, Children’s Cancer Hospital, Cairo, Egypt; 5 Department of Physiology, Faculty of Veterinary Medicine, Suez Canal University, Ismailia, 41522 Egypt; 6 Urology and Nephrology Center, Mansoura University, Mansoura, Egypt; 7 Center of Excellence for Stem Cells and Regenerative Medicine (CESC), Zewail City of Science and Technology, Sheikh Zayed District, Giza 12588, 6th of October City, Egypt

**Keywords:** Adipose stem cells, Angiogenesis, Diabetes mellitus, Diabetic serum, Diabetic patients, Metabolic activity, Type 2 diabetes

## Abstract

**Background:**

Type 2 diabetes is an endocrine disorder characterized by compromised insulin sensitivity that eventually leads to overt disease. Adipose stem cells (ASCs) showed promising potency in improving type 2 diabetes and its complications through their immunomodulatory and differentiation capabilities. However, the hyperglycaemia of the diabetic microenvironment may exert a detrimental effect on the functionality of ASCs. Herein, we investigate ASC homeostasis and regenerative potential in the diabetic milieu.

**Methods:**

We conducted data collection and functional enrichment analysis to investigate the differential gene expression profile of MSCs in the diabetic microenvironment. Next, ASCs were cultured in a medium containing diabetic serum (DS) or normal non-diabetic serum (NS) for six days and one-month periods. Proteomic analysis was carried out, and ASCs were then evaluated for apoptosis, changes in the expression of surface markers and DNA repair genes, intracellular oxidative stress, and differentiation capacity. The crosstalk between the ASCs and the diabetic microenvironment was determined by the expression of pro and anti-inflammatory cytokines and cytokine receptors.

**Results:**

The enrichment of MSCs differentially expressed genes in diabetes points to an alteration in oxidative stress regulating pathways in MSCs. Next, proteomic analysis of ASCs in DS revealed differentially expressed proteins that are related to enhanced cellular apoptosis, DNA damage and oxidative stress, altered immunomodulatory and differentiation potential. Our experiments confirmed these data and showed that ASCs cultured in DS suffered apoptosis, intracellular oxidative stress, and defective DNA repair. Under diabetic conditions, ASCs also showed compromised osteogenic, adipogenic, and angiogenic differentiation capacities. Both pro- and anti-inflammatory cytokine expression were significantly altered by culture of ASCs in DS denoting defective immunomodulatory potential. Interestingly, ASCs showed induction of antioxidative stress genes and proteins such as SIRT1, TERF1, Clusterin and PKM2.

**Conclusion:**

We propose that this deterioration in the regenerative function of ASCs is partially mediated by the induced oxidative stress and the diabetic inflammatory milieu. The induction of antioxidative stress factors in ASCs may indicate an adaptation mechanism to the increased oxidative stress in the diabetic microenvironment.

**Supplementary Information:**

The online version contains supplementary material available at 10.1186/s13098-024-01365-1.

## Background

Diabetes mellitus is a metabolic disorder caused by defective glucose tolerance resulting from impaired insulin secretion (type 1 diabetes), defective insulin action (type 2 diabetes), or both [1]. In the more common type 2 diabetes, lack of tissue sensitivity to insulin and impaired regulation of glucose production lead to impaired β-cell functionality and, eventually, β-cell dysfunction [2, 3].

Stem cell therapy provides a promising treatment for type 2 diabetes. Mesenchymal stromal cells (MSCs) have shown beneficial effects in treating diabetes in animal models and in clinical trials [4–6]. Adipose tissue derived MSCs display superior proliferative potential, diverse differentiation capacities, and immunosuppressive qualities, supporting their usage in regenerative therapy [7–9]. Importantly, ASCs are easily accessible due to the availability of adipose tissue, making them a premium source of autologous stem cell transplants [10]. ASCs were shown to reduce type 2 diabetes complications via promoting angiogenesis, reducing inflammation, and inducing tissue repair [11–15]. ASCs efficiently promoted wound healing associated with type 2 diabetes [16]. This effect was due to the enhancement of vascularization in the wound area [8, 16]. In diabetic rats, ASCs were reported to reduce ischemia and restore erectile dysfunction through promoting angiogenesis and neovascularization in the ischemic limb [12, 15, 17]. In clinical studies, ASCs showed encouraging results by inducing bone repair in type 2 diabetic patients [18, 19]. The ability of MSCs to differentiate into adipocytes was found to play a role in skin regeneration and wound healing, rendering them especially promising for diabetic complications [20, 21].

Successful cellular therapy using ASCs in type 2 diabetic patients necessitates an understanding of how the patient’s diabetic milieu interacts with and impacts the transplanted healthy cells. In vitro data showed that hyperglycemia deteriorated the regenerative capacities of MSCs by impairing their viability, compromising their differentiation and angiogenic potential, and promoting DNA damage [22, 23]. Transcriptomic analysis of MSCs isolated from diabetic patients showed changes in their immunomodulatory markers and crucial inflammatory signaling pathways, all of which may contribute to impaired therapeutic potential [24].

Since ASCs are among the most widely used type of stem cells in clinical trials for type 2 diabetes, understanding the alterations in their function in the diabetic microenvironment will impact and further refine their utility [5]. Despite their wide-spread use in the clinic, few studies looked into the altered regenerative potential of ASCs after living in the patient’s diabetic microenvironment. This microenvironment surrounds the diabetic patients’ cells bathing in hyperglycemic extracellular fluid [25–27] In this study, we used serum from type 2 diabetic patients to replicate the in vivo cellular microenvironment of the type 2 diabetic patients [28, 29]. The outcome of this study should provide new insights to ultimately enhance the culture conditions of ASCs prior to their administration for the treatment of diabetic patients.

## Materials and methods

### Data collection and functional enrichment analysis of MSCs differentially expressed genes (DEGs) in the diabetic microenvironment

In the PUBMED database, we conducted literature mining to search for the significant and differential gene expression profile of MSCs in the diabetic microenvironment (*p* < 0.05; fold change > 1.5). We used the keywords (mesenchymal stem cell) AND (type 2 diabetes OR diabetic microenvironment) AND (genes OR gene expression profile OR gene expression array). All diabetic differentially expressed genes (DEGs) were merged and then integrated to undergo subsequent enrichment analysis.

The functional enrichment analysis for the obtained DEGs was performed using Enrichr-computational systems biology [amp.pharm.mssm.edu/Enrichr/]. Enrichr provides the most enriched gene ontology (GO) terms and KEGG pathways, offering the most relevant functions in which a certain gene list can be involved. The most enriched pathways, extracted from Enrichr by filtration of *p* < 0.05, were then deeply studied to predict the altered pathways in MSCs in the diabetic microenvironment.

### Ethical statement and patients selection

All methods were carried out in accordance with guidelines and regulations of the approved protocol by the ethical committee of Kasr Alainy, Faculty of Medicine, Cairo University. The ethical approval number is N-55-2019. Blood samples were collected following informed consent from diabetic patients and healthy volunteers. Fifteen patients had type 2 diabetes mellitus and satisfied the American Diabetes Association criteria with no history of diabetes complications. Diabetic participants who received thiazolidinedione were excluded from the study since the drug affects the osteogenic differentiation of ASCs. Exclusion criteria also included pregnant females, smokers, alcoholics, patients receiving hormone replacement therapy, thyroid disease, or other known medical conditions. The non-diabetic normal serum was collected from 15 non-diabetic normoglycemic, age- and gender-matched participants who were not receiving medications.

### Serum isolation and MSC culture

Venous blood samples from diabetic patients and healthy subjects were collected into vacutainers containing no additives. Blood samples from diabetics and healthy participants were left to clot and then centrifuged for 10 min at 2000 ×g at 4 °C. The normal and diabetic sera were then pooled, filtered, and stored at − 20 °C.

### Cell culture and study groups

ASCs were a kind gift from the Urology and Nephrology Center, Mansoura University, Egypt (characterized in supplementary Fig. 1). ASCs of passages 3–5 were assigned to an experimental and a control group. Samples from the experimental group were cultured in DMEM with 10% of diabetic serum (DS), and the control group was cultured in DMEM with 10% of normal serum (NS). ASCs were cultured in either DS or NS for short-term culture (6 days) or long-term culture (one month).

### Flow cytometry characterization

Phenotypic characterization was conducted for ASCs cultured for one month and six days in DS and NS using flow cytometry, following the surface markers recommended by the International Society of Stem Cells [30]. A blocking buffer of 1% BSA was added to ASCs for 10 min. Cells were then centrifuged, resuspended in the blocking buffer at a concentration of 1 × 10^6^ cells/mL, and stained with the following monoclonal antibodies for 30 min. FITC anti-CD90 and PerCP-Cy anti-CD105 monoclonal antibodies (BD Bioscience, 560,819), and hematopoietic stem cell markers; APC anti-CD45 monoclonal antibodies (BD Bioscience, 555,485). Unstained samples were used as a negative control for gating. Flow cytometry was conducted using FACSCalibur™ (Becton DickiNSon) following standard procedures using CellQuest Pro Software (Becton DickiNSon). Data analysis was processed using FlowJo v. 10.2 software (Treestar, USA) with super-enhanced Dmax (SED) subtraction analysis to detect differences in histograms. Experiments were carried out in triplicate.

### RNA extraction and real-time PCR

Total RNA was extracted from ASCs cultured in DS and NS after 6 days and one month using TRIZOL RNA isolation kit (Thermo Fisher Scientific, USA) according to the manufacturer’s protocol. The total RNA was transcribed into cDNA using the Revertaid first-strand cDNA synthesis Kit (Thermo Fisher Scientific, USA). Real time PCR was carried out using 150 ng of a cDNA template of purity between 1.7 and 2.0 and SsoAdvanced™ Universal SYBR® Green Supermix (Bio-Rad, USA) was used. Samples were then amplified using the CFX96 Touch™ Real-Time PCR Detection System (Bio-Rad, USA).The relative gene expression was normalized to the β-actin gene and calculated using the comparative threshold (2^ΔΔCT^) method [31]. Experiments were performed in triplicate (see **Primer List** for primer sequences).

### Apoptosis assay

Apoptosis assay was carried out for ASCs cultured in DS and NS after 6 days and one month according to the manufacturer’s instructions. Apoptosis was measured for ASCs at a concentration of 1 × 10^6^ cells/mL using the Annexin-V-FITC and propidium iodide (PI) apoptosis detection kit (Miltenyi Biotec Inc., USA). FACSCalibur™ (Becton DickiNSon) was used following standard procedures using CellQuest Pro Software (Becton DickiNSon), and data analysis was processed using FlowJo v. 10.2 software (Treestar, USA) [32].

### Scratch assay

Scratch assay was used to test the migratory capacity of cells under diabetic conditions as described [33, 34]. The ASC monolayers with an initial seeding density of 1 × 10^4^ cells/mL were cultured in either DS, NS, or fetal bovine serum (FBS) as a control for 6 days. Cells were incubated untill they reached 80% confluency. Before starting the assay, cell monolayers were cultured in serum-free medium for 24 h. ASC cultures were scratched with a pipette tip, then washed with PBS to remove cell debris, and then cultured in serum free medium. Using ultrafine tip markings on the other side of the plate, reference points were made. Cells were photographed with phase-contrast microscopy at the same reference points at 0 and 24 h. ImageJ 1.44P was used to quantitatively evaluate the gap distance.

### Differentiation assay

Adipogenic and osteogenic differentiation were tested for ASCs cultured in DS and NS on day 6 [35]. For osteogenic differentiation, ASCs were cultured in a complete culture medium supplemented with 100 nM dexamethasone, 100 µM ascorbic-2-phosphate, and 100 mM glycerol-phosphate (Sigma Aldrich) for 21 days. Alizarin red staining was used to visualize the calcium nodules and confirm osteogenic differentiation. Adipogenic differentiation was induced by the culture of ASCs in a complete culture medium supplemented with 78 µl dexamethasone, 1250 µl bovine insulin, 62.5 µl indomethacin, and 55.6 µl of 3-isobutyl-1-methylxanthine for 14 days. Oil-red O staining was used to detect oil droplet vacuoles and confirm adipogenic differentiation.

### Angiogenesis assay

ASCs were cultured in DS and NS for 6 days and one month and were evaluated for angiogenic differentiation using an angiogenesis starter kit (Gibco, USA) according to the manufacturer’s instructions. Briefly, Geltrex® LDEV-free reduced growth factor basement membrane matrix (Invitrogen, USA) was thawed at 4°C, used to coat a 24-well plate (100 µl/well), and incubated at 37 °C for 30 min until solidification. ASCs were seeded at density 3 × 10^4^ cells/ml medium (large vessel endothelial-supplemented medium 200, Gibco, USA) and incubated overnight at 37° C in a humidified atmosphere of 5% CO^2^. After 16 h, ASCs were stained with Calcein AM (2 µg/mL, Molecular Probes, USA), incubated for 30 min at 37 °C with5% CO2, and then imaged using an inverted fluorescent microscope (Leica DMi8, Leica Microsystems, Germany). Tube formation was evaluated by measuring the total number of branching points in 3 photographic fields of each well. The assay was performed in triplicate [35, 36].

### Oxidative stress detection

To detect intracellular ROS, a DCFDA (dichloro-dihydro-fluorescein-diacetate) assay was performed using the DCFDA/H2DCFDA Cellular ROS Assay Kit (Abcam, USA) for ASCs cultured in DS and NS for 6 days and one month, according to the manufacturer’s protocol. ASCs were seeded at a density of 25 × 10^3^ cells/ml and grown in 96-well plates overnight. Cells were washed with buffer and incubated with DCFDA solution for 45 min at 37 °C in the dark. DCFDA was detected using FLUOstar Omega microplate reader (BMG LABTECH, Ortenberg, Germany) at Ex/Em = 485 and 535 nm [37]. Data were analysed using MARS data analysis software v.3.20 R2.

### Shotgun proteomics analysis

#### Preparation of cell protein lysate

Collected ASCs were rinsed with PBS and centrifuged at 10,000 rpm. A protein extract of cells was obtained by placing approximately 20 µl of lysis solution (8 M urea, 500 mM Tris HCl, pH 8.5) with complete ultra-proteases and phosphatase inhibitors (Roche, Mannheim). Samples were incubated at 37° C for 1 h with occasional vortexing, then centrifuged at 12,000 rpm for 20 min. The lysate was assayed using the BCA method (Pierce, Rockford IL) at Å562 nm before digestion.

#### Proteins tryptic digest

30 *µ*g of cell protein lysate from each sample were subjected to in-solution digestion. In brief, protein pellets were re-suspended in an 8 M urea lysis solution and reduced with 200 mM 1,4-Dithiothreitol (DTT) for 30 min. Alkylation of cysteine residues was performed using 10 mM iodoacetamide for 30 min in a dark area. Samples were diluted to a final concentration of 2 M urea with 100 mM Tris-HCl, pH 8.5, before digestion with trypsin. For endopeptidase digestion, modified procaine trypsin (Sigma, Germany) was added at 30:1 (protein: protease mass ratio) and incubated overnight in a thermo-shaker at 600 rpm at 37 °C. The digested peptide solution was acidified using 90% formic acid to a final pH of 2.0. The resultant peptide mixture was cleaned up using a stage tip, as discussed earlier [38]. Peptides were assayed using the BCA method (Pierce, Rockford IL) at Å562 nm before injection (1ug/10ul).

#### Nano-LC MS/MS analysis

Nano-LC MS/MS analysis was performed using TripleTOF 5600 + (AB Sciex, Canada) interfaced at the front end with Eksigent nanoLC 400 autosamplers with an Ekspert nanoLC 425 pump. Peptides were trapped on CHROMXP C18CL 5 μm (10 × 0.5 mm) (Sciex, Germany) in trap and elute mode. MS and MS/MS ranges were 400–1250 m/z and 170–1500 m/z, respectively. A design of a 120-minute liner gradient 3–80% solution (80% ACN, 0.2% formic acid). The 40 most intense ions were sequentially selected under data-dependent acquisition (DDA) mode with a charge state of 2–5. For each cycle, survey full scan MS and MS/MS spectra were acquired at a resolution of 35.000 and 15.000, respectively. To ensure accuracy, external calibration was scheduled and run during sample batches to correct possible TOF deviation. Each sample was run in triplicate.

#### Proteomics data analysis

Raw LC/MS/MS data in Wiff format from the TripleTOFTM 5600 + were searched using Protein Pilot software (version 5.0.1.0, 4895) with the paragon Algorithm (version 5.0.1.0, 4874). Trypsin was used as a digestion factor for the peptides identified from MS/MS spectra, and then the Pro Group™ Algorithm assembles peptide identifications into a list of reliable protein identifications. Iodoacetamide was selected as the Cys alkylation and urea denaturation as the special factors used in the experiment. Uniprot *Homosapiens* (Swiss-Prot and TrEMBL databases containing 224,139 proteins) was used for searching. Analysis was searched with Bias Correction. T-s false discovery rate (FDR) was kept at 1% at the protein level to assure high quality.

### Statistical analysis

Data were analysed using the statistical package SPSS version 16 and summarized using the mean and standard deviation. Comparisons between groups were conducted using the independent t-test and ANOVA for normally distributed quantitative variables. In contrast, Kruskal-Wallis and Mann-Whitney tests were used when data were non-parametrically distributed. A correlation was done to test for linear relations between quantitative variables using the Pearson coefficient. Graphs were prepared using GraphPad Prism (GraphPad Software, San Diego, CA, USA), and figure alignment was prepared using BioRender online software. Significance was indicated at * *P* < 0.05, ** *P* < 0.01, *** *P* < 0.001, and **** *P* < 0.0001. Before proteomic analysis, a probabilistic quotient normalization (PQN) was applied using a reference sample (NS1-1). Proteins with NAs in two or more samples were removed from further analysis. A pair-wise approach was used to examine the signature of proteins in this study (i.e., DS1 vs. NS1, DS6 vs. NS6, and DS1 vs. DS6). Then, a fold change (FC) analysis was performed for each pair-wise analysis on the proteins found in all groups with two FC thresholds. Pathway enrichment analysis and gene ontology annotation were done with g: Profiler with a p-value less than 0.05 and FDR 5% and drawn with R. GO analysis was applied to the significant proteins from the FC calculations and unique proteins for each group (please check https://biit.cs.ut.ee/gprofiler/gost). ​

## Results

### Bioinformatics functional enrichment and proteome analysis for ASCs cultured in diabetic microenvironment

**Fig. 1:**
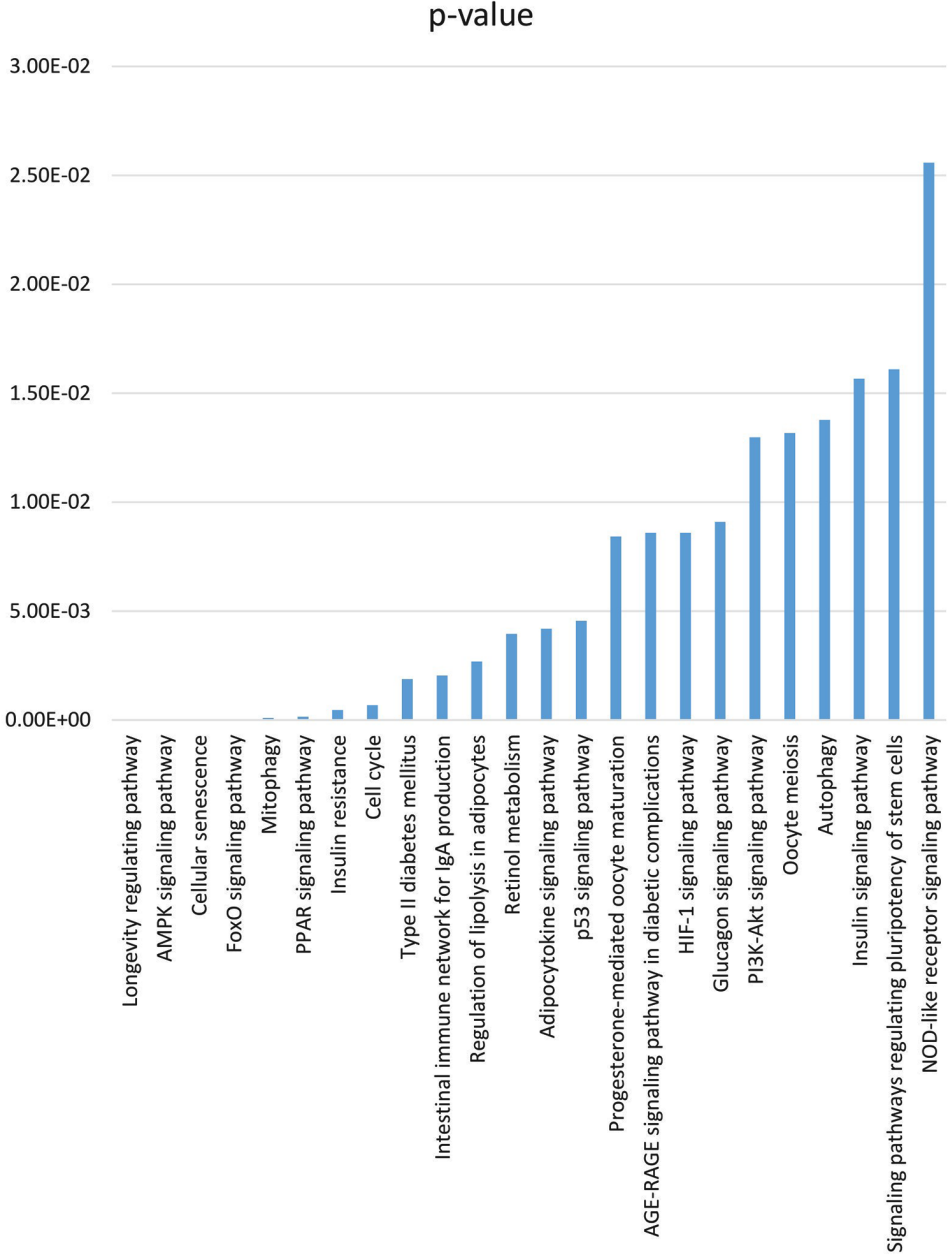
Enriched pathways (KEGG) for the MSC dysregulated genes in diabetic microenvironment.

To start with, KEGG, Wikipath, and Reactomepathway enrichment analysis were conducted to predict the effect of the diabetic microenvironment on the functionality and characteristics of MSCs. The analysis indicated alteration of many metabolic pathways related to diabetes, such as insulin and glucagon signaling pathways and regulation of lipolysis in adipocytes. Moreover, pathways that control cellular senescence, autophagy, and mitophagy, as well as the signaling pathways that regulate pluripotency of stem cells, have been significantly enriched (Fig. 1 and Supplementary Table 1), indicating an alteration in the stem cell properties and regenerative capacity of ASCs cultured in diabetic serum. The enrichment of MSC DEGs in diabetes also showed alteration in oxidative stress regulating pathways, such as the AGE-RAGE signaling pathway, FoxO, HIF-1 signaling pathways, and adipocytokine pathway.

**Fig. 2:**
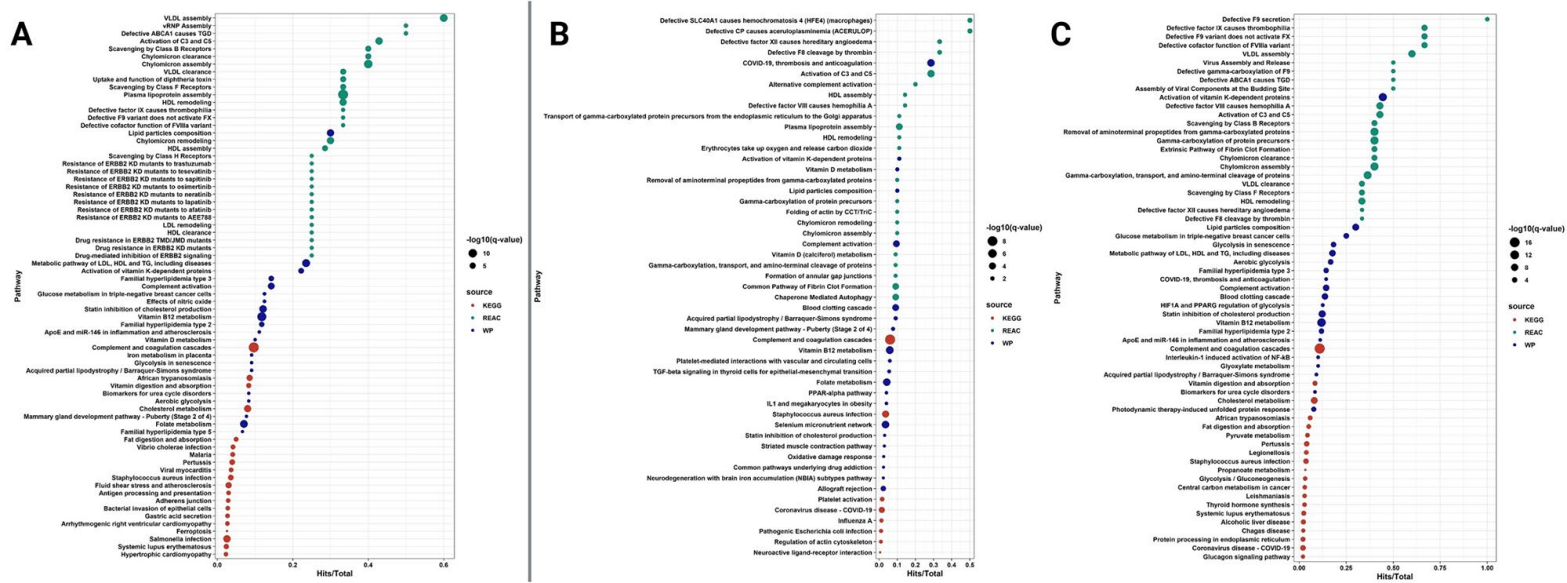
A-C: Selected Pathway enrichment analysis (based on FC) with FDR 5% (q-value) and p-value  >  0.05 using different background databases. A) represents DS6 vs NS6 samples B) represents DS1 vs NS1. C) represents DS1 vs DS6 samples. Each database has a color code illustrated in the figure legend. The size of the circle reflects the number of -log10 (q-value). The x-axis is the –Hits/total ratio. This analysis includes up- and down-regulated proteins with a fold changecut-off point of 2.

**Fig. 3:**
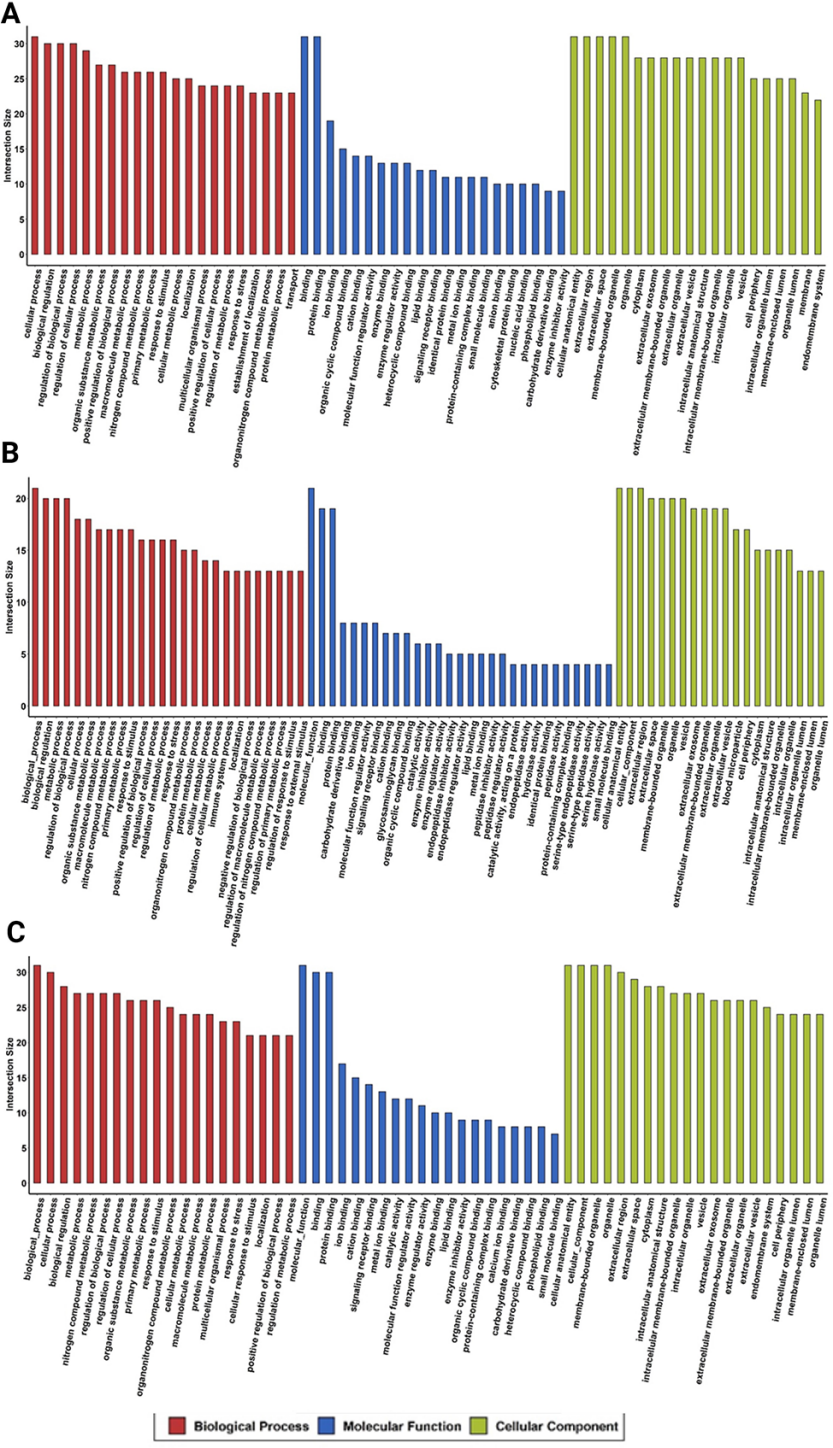
A-C: GO FDR 0.05 (based on FC): Selected Gene ontology enrichment annotation analysis was performed on the significant proteins from the FC calculations, with a p-value less than 0.05 and FDR 5%. A) represents DS6 vs NS6 samples B) represents DS1 vs NS1. C) represents DS1 vs DS6 samples. The top 20, based on the protein intersection size from each GO category were plotted.

Next, we conducted proteomic analysis of ASCs grown in normal or diabetic serum that showed dramatic differences in their proteome (qualitatively and on relative quantitation). In brief, 2176 proteins were reported in total. Among them, 509, 649, 1246, and 885 proteins were reported for DS1, DS6, NS1, and NS6 (Supplementary Table 2, supplementary Figs. 2–3). Relative quantitation using the normalized spectral abundance factor (NSAF) presents the relative abundance of identified proteins between the experimental groups (Supplementary Tables 3–5). As demonstrated in Figs. 2 and 3, differentially expressed proteins were shown to be up or downregulated in the diabetic group compared to the normal group. Supplementary Tables 6–11 show selected pathway enrichment analyses. Data showed differentially expressed proteins involved in pathways related to cell differentiation, apoptosis, and oxidative stress (Supplementary Tables 6–11). Based on these data, we further experimentally tested the regenerative capacity of ASCs in a diabetic microenvironment.

### ASCs displayed altered morphology after long-term culture in the diabetic microenvironment

After 6 days, ASCs cultured in DS started to show morphological signs of cellular aging [39]. ASCs became more flattened, acquired a granular surface, and their cytoplasm became vacuolated. After one month of culture in DS, the cells became rounded and detached, and cell confluency decreased. Similar changes were observed in cells cultured in NS, but to a much lower extent (Supplementary Fig. 4).

### The diabetic microenvironment induced phenotypic changes in ASCs after long-term culture

**Fig. 4:**
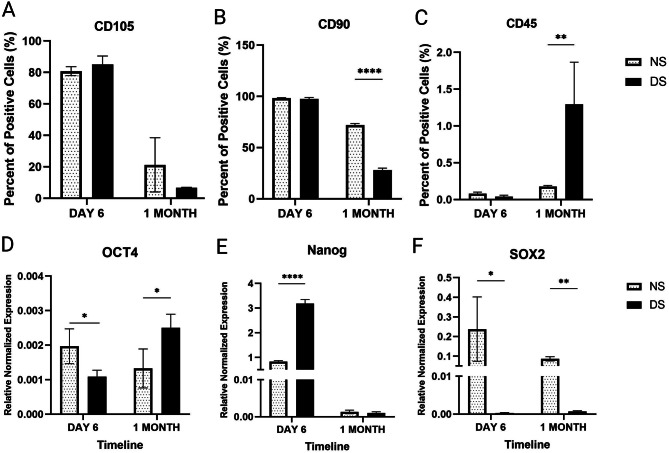
A-F: A-C) Phenotypic characterization for ASCs cultured in diabetic microenvironment; diabetic serum (DS), normal serum (NS) after 6 days and one month using flowcytometry. D-F) The expression of pluripotency markers in ASCs after 6 days and one-month culture in DS and NS using QPCR. Data are represented as the means ± SD. Asterisks indicate a difference compared with the control non treated group. *P  >  0.05, ** P  >  0.01, **** P  >  0.0001.

ASCs isolated from diabetic patients showed an alteration in their surface markers, which may reduce their immunosuppressive and differentiation potential [40–44]. Thus, we examined for surface marker changes in ASCs following culture in DS. There were no significant differences in the expression of CD90 between ASCs cultured in NS and DS after 6 days of culture (*P* = 0.77). However, after one month, the expression of MSC marker CD90 in cells cultured in DS was significantly lower than those cultured in NS (2.6-fold, *P* < 0.0001). There were no significant changes, however, in CD105 expression (*P* > 0.05). Expression of the hematopoietic marker CD45 was significantly higher for ASCs cultured in DS for one month compared to those cultured in NS (7.3-fold, *P* = 0.004) (Fig. 4A-C).

### The diabetic microenvironment induced changes in ASC pluripotency markers

We then examined the expression of pluripotency markers since they control the cell cycle, proliferation, and differentiation of MSCs [45, 46]. The expression of SOX2 in DS-treated cells was significantly lower than that of those cultured in NS at 6 days and one month (*P* = 0.05 and *P* < 0.01, respectively, Fig. 4D-F). Also, OCT4 expression was significantly lower in DS than in NS-treated cells (*P* < 0.05) on day 6. However, this low expression was reversed after one month of culture, where cells treated with NS showed significantly lower OCT4 expression than DS- treated cells (*P* < 0.05, Fig. 4D-F). On the contrary, the expression of Nanog was significantly higher in DS-treated cells compared to NS-treated cells on day 6 (*P* < 0.0001, Fig. 4D-F), but not at one month.

### The diabetic microenvironment accelerated the apoptotic rate of ASCs and induced their intracellular oxidative stress

**Fig. 5:**
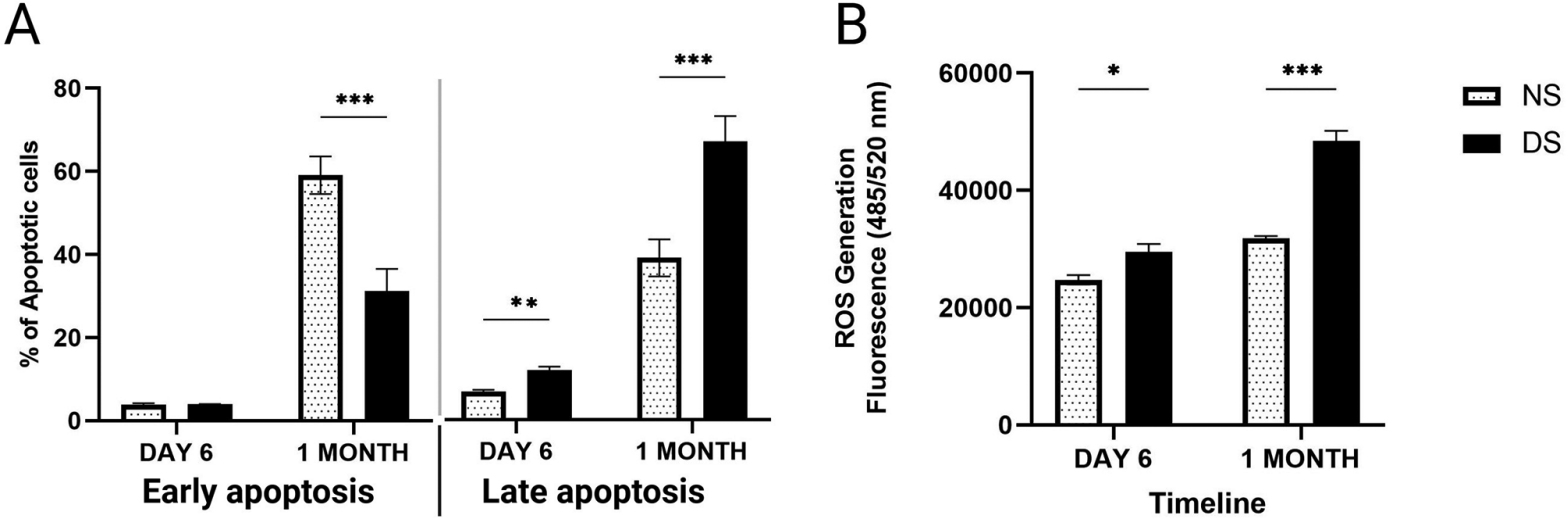
A-B: A) Apoptosis assay for ASCs cultured in normal serum (NS) and diabetic serum (DS). B) DCFDA assay for ASCs cultured in DS and NS for 6 days and 3 weeks. Data are represented as the means ± SD. Asterisks indicate a difference compared with the control non treated group. *P  >  0.05, **P  >  0.01, ***P  >  0.001

To investigate the cytotoxic effect of the diabetic microenvironment on ASCs, we used serum concentrations of 10% and 20%, in DS and NS for 1, 2, 6 days, and one month. No significant changes in apoptosis of cells cultured in DS or NS were observed after 2 days, regardless of the serum concentration (results not shown). Early apoptotic changes of ASCs (after 6 days of culture) were non-significant in DS compared to NS (*P* > 0.05). Late apoptosis, on the other hand, was considerably higher in cells cultured in DS compared to those cultured in NS (1.8-fold, *P* < 0.01) after 6 days of culture. Maintaining the cells in either NS or DS for one month significantly increased early apoptosis (15-fold, *P* = 0.0001 and 7.9-fold, *P* = 0.001, respectively, Fig. 5A). Late apoptosis was significantly higher in cells cultured for one month in DS compared to NS (1.7-fold, *P* < 0.001).

Intracellular ROS levels showed a significant increase in ASCs after 6 days and one-month of culture in DS compared to those cultured in NS (Fig. 5B).

### Migration capacity of ASCs cultured in diabetic serum

**Fig. 6:**
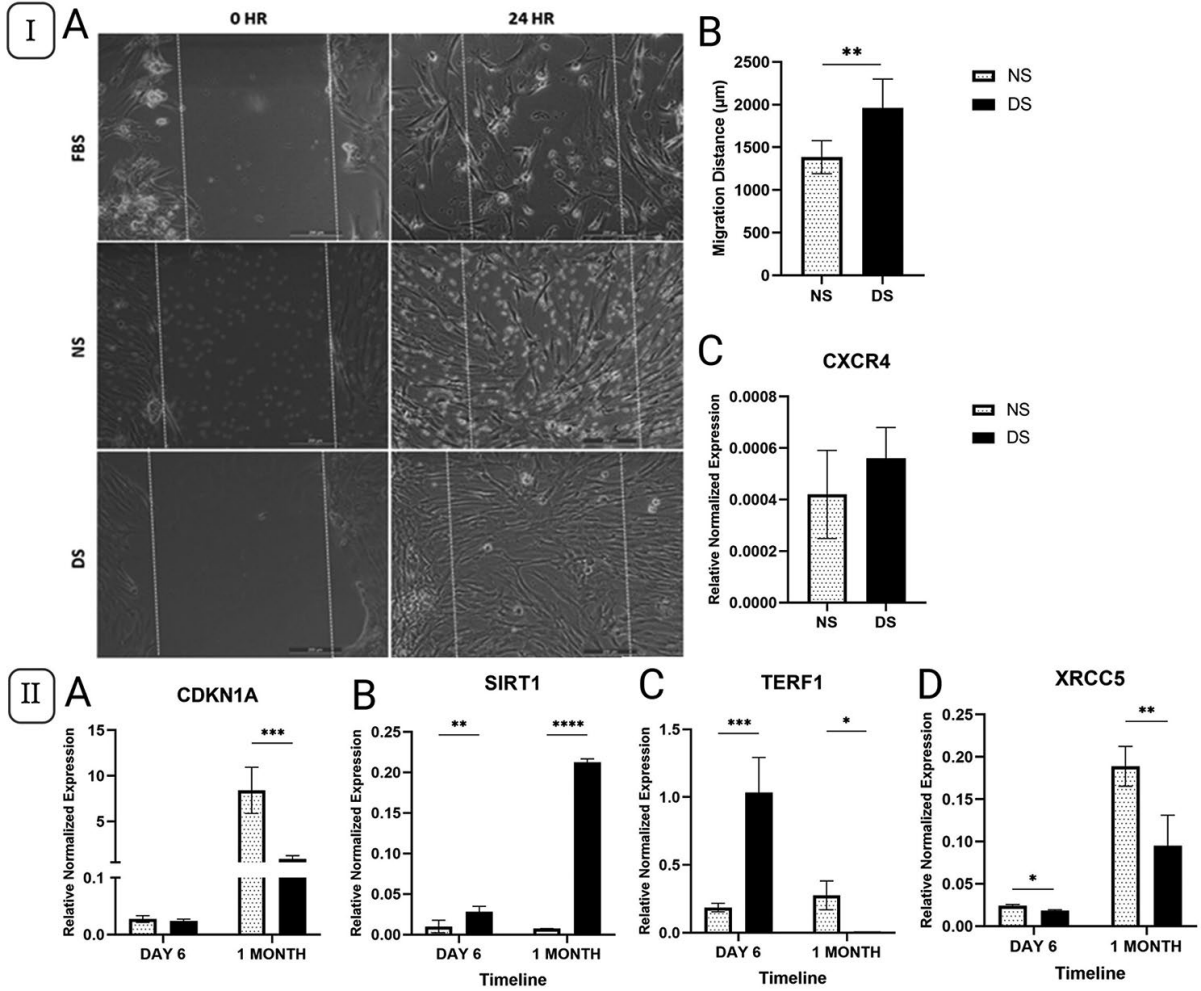
I-II: (I) A-B) Scratch assay analysis of the migration capacity for ASCs cultured in diabetic serum (DS) in comparison to normal serum (NS), FBS was used as a negative control after 6 days of culture. C) The expression of migration related marker; CXCR4 in ASCs after 6 days in DS and NS using QPCR. (II) The expression of DNA repair markers (XRCC5, TERF1, CDKN1A and SIRT1) in ASCs after 6 days and one-month culture in DS and NS using QPCR. Data are represented as the means ± SD. Asterisks indicate a difference compared with the control non treated group. *P  >  0.05, ** P  >  0.01, ***P  >  0.001, **** P  >  0.0001.

On day 6, the migration of ASCs was increased after treatment with DS compared to NS (*P* = 0.0028, Fig. 6I), as determined by scratch assay. However, the expression of CXCR4, a gene involved in cell migration, showed no significant difference between cells cultured in DS or NS at the same time point (*P* > 0.05, Fig. 6I).

### The diabetic microenvironment reduced the expression of DNA repair markers in ASCs

Hyperglycemia enhances DNA damage in MSCs; hence, we assessed the expression of DNA repair markers XRCC5, TERF1, CDKN1A, and SIRT1 in ASCs in a diabetic milieu [23]. The expression of XRCC5 was significantly lower in cells cultured in DS compared to NS at both time points (*P* < 0.05 and *P* < 0.01, respectively). The expression of TERF1 was significantly higher in cells cultured in DS compared to NS after 6 days (*P* < 0.001). However, its expression levels decreased in DS compared to NS (*P* < 0.05) after one month. The expression of CDKN1A did not show any significant difference after 6 days of culture in DS or NS (*P* > 0.05) but was significantly lower in DS at one month (*P <* 0.001, Fig. 6 II). DS-treated cells showed substantially higher SIRT1 gene expression compared to NS-treated cells in both short-term and long-term cultures (*P* < 0.01 and *P* < 0.0001, respectively, Fig. 6 II).

**Fig. 7:**
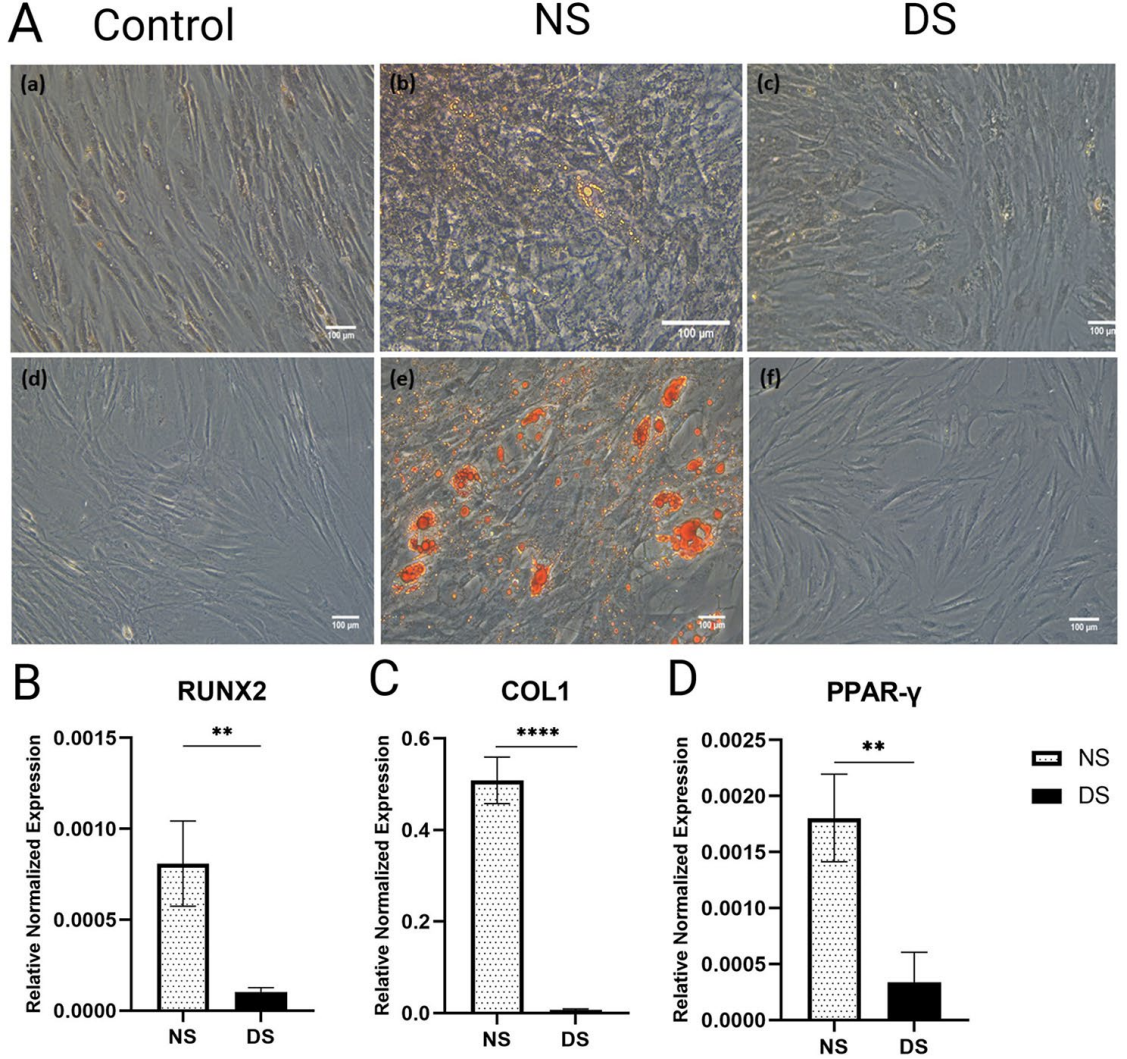
A-D: Osteogenic and adipogenic differentiation assays for ASCs cultured in diabetic serum (DS) in comparison to normal serum (NS). A) Decreased osteogenic (a-c) and adipogenic differentiation (d-f) for ASCs cultured in DS, and NS after 6 days of culture. B-D) QPCR analysis for the osteogenic markers; RUNX2 and COL1 (B, C) in addition to the adipogenic marker PPARγ (D). Data are represented as the means ± SD. Asterisks indicate a difference compared with the control non treated group. **P  >  0.01, **** P  >  0.0001.

### Adipogenic and osteogenic lineages differentiation of ASCs were compromised in the diabetic microenvironment

We investigated the differentiation capacity of ASCs after 6 days of culture in DS and NS into adipogenic and osteogenic lineages as determined by oil red O and alizarin red stains, respectively (Fig. 7A). Morphologically, there was a noticeable decrease in cytoplasmic oil droplets formed in ASCs cultured in DS compared to those cultured in NS, and also a decrease in calcium deposits stained by Alizarin red. There was a significant concomitant decrease in Runx2 and Col1 osteogenesis markers, as shown by qPCR. (*P* < 0.01 and *P* < 0.0001, respectively) (Fig. 7B-C). Similarly, there was a decrease in the adipogenic marker ${\rm{PPARy}}$ after 6 days of culture in DS (*P* < 0.01, Fig. 7D).

### Angiogenic differentiation of ASCs was altered in the diabetic microenvironment

**Fig. 8:**
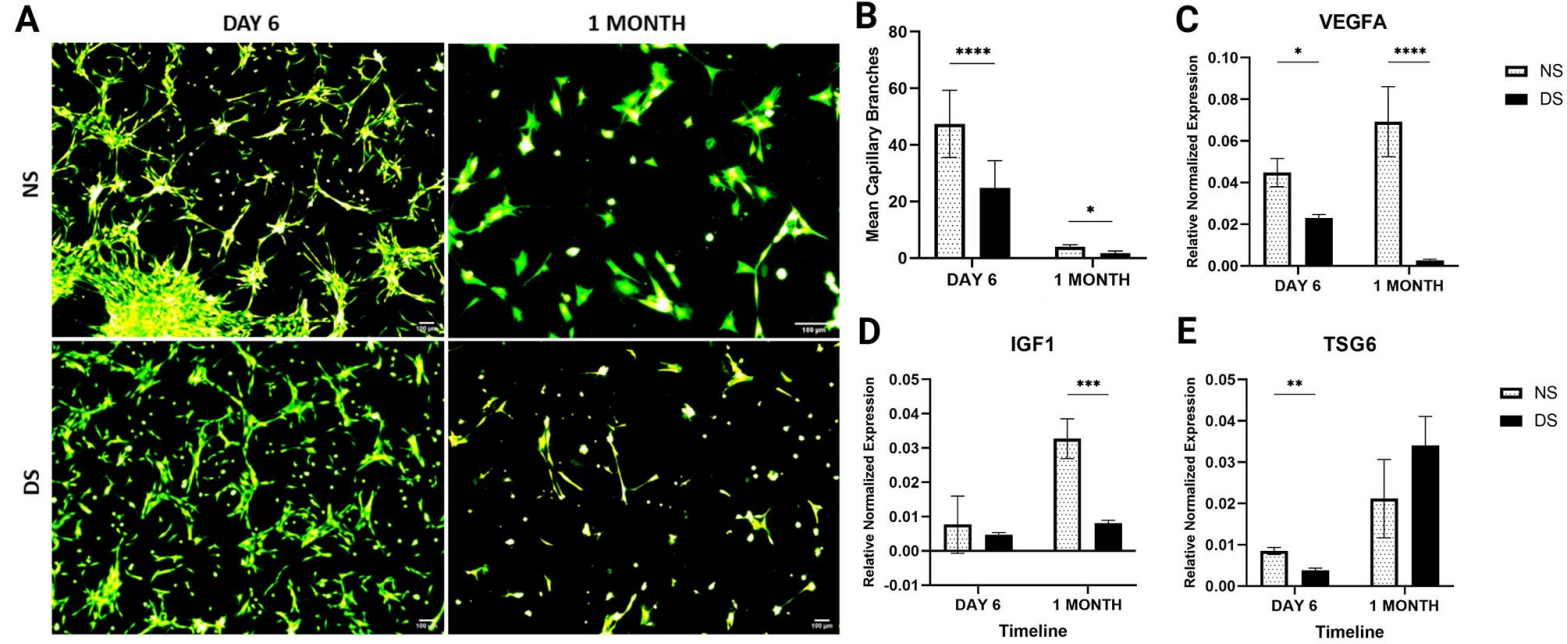
A-E: A-B) Tube formation assay for the angiogenesis capacity of MSCs in diabetic microenvironment. C-E) Angiogenesis marker expressions (VEGFA, TSG6 and IGF1) were measured using QPCR. Data are represented as the means ± SD. Asterisks indicate a difference compared with the control non treated group. *P  >  0.05, ** P  >  0.01, ***P  >  0.001, **** P  >  0.0001.

ASCs showed markedly fewer tube formations in the angiogenesis assay and shorter malformed branches after 6 days in DS (*P* < 0.0001, Fig. 8A-B). After one month in DS, cells displayed more pronounced shortening and disaggregated branch formation compared to those cultured in NS (*P* < 0.05). The expression of angiogenesis markers VEGFA and TSG6 was significantly decreased after 6 days in culture in DS (1.95-fold, *P* = 0.05, and 2.2-fold, *P* < 0.01, respectively). However, there was no change in the expression of the IGF1 angiogenesis marker during the same period (*P* > 0.05). After one month, the expression of VEGFA and IGF1 decreased significantly in cells cultured in DS compared to NS (25-fold, *P* < 0.0001 and 4-fold, respectively, *P* < 0.001, Fig. 8C-E).

### Inflammatory marker expression was altered in the diabetic microenvironment

**Fig. 9:**
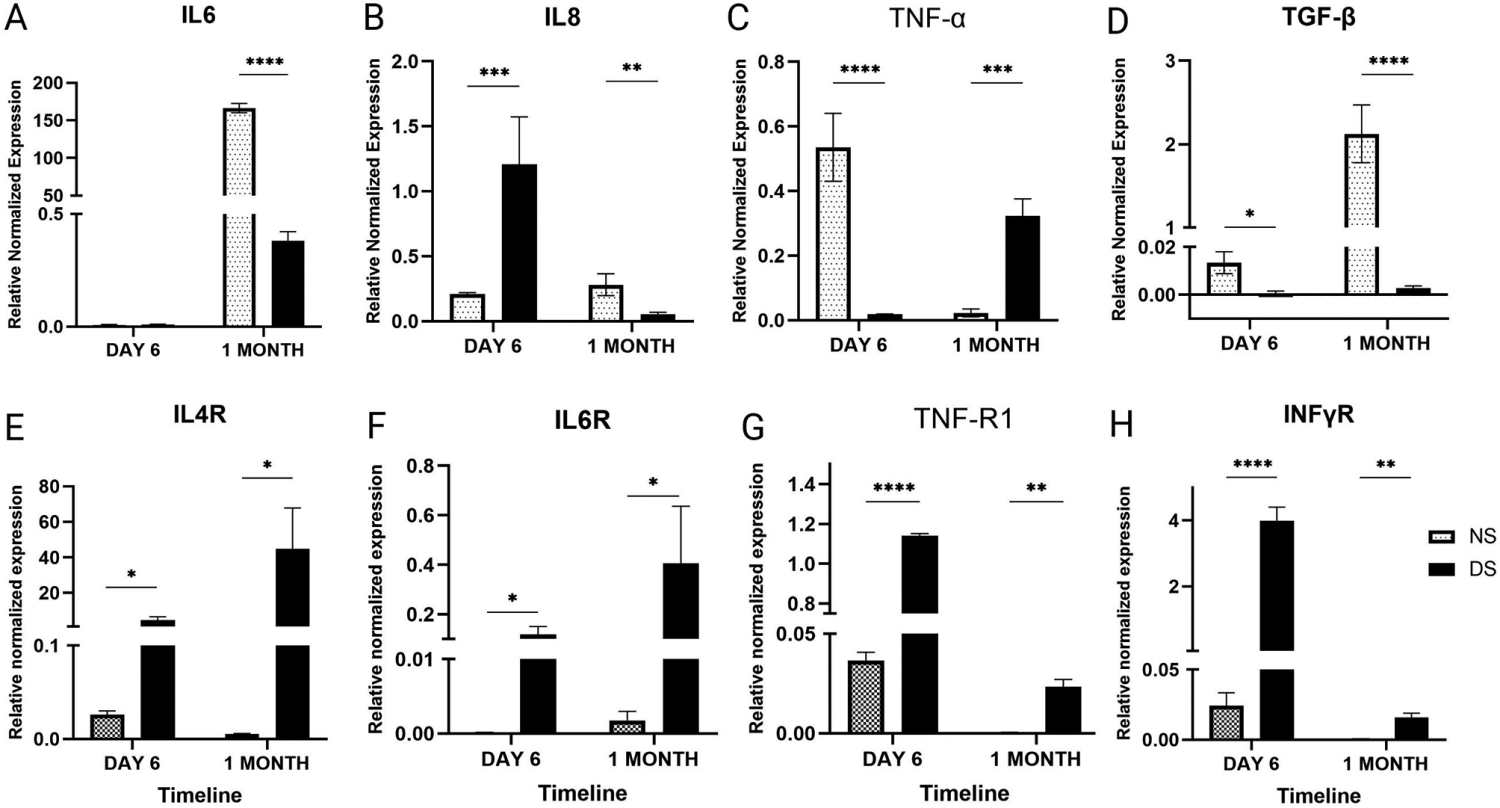
A-H: A-D) The expression of inflammatory markers (IL-8, TSG6, TNFα, IL-6, TGFβ) in ASCs cultured in DS and NS after 6 days and one month. E-H) The expression of cytokine receptors on ASCs (IL-6R, IL-4R, INFγR and TNF-R1). Data are represented as the means ± SD. Asterisks indicate a difference compared with the control non treated group. *P  >  0.05, ** P  >  0.01, ***P  >  0.001, **** P  >  0.0001.

The expression of IL-6 showed no significant change after 6 days, while TNFα and TGF-β expression were significantly lower in DS compared to NS-treated cells (29.5-fold, *P* < 0.0001, and 23.5-fold *P* < 0.05, respectively). On the other hand, IL-8 expression was significantly higher in DS compared to NS-treated cells (5.8-fold, *P* < 0.001). After one month of culture, IL-6, IL-8, and TGF-β expressions were all significantly lower in DS- treated cells compared to NS (435-fold *P* < 0.0001, 5-fold *P* < 0.01, and 781.9-fold *P* < 0.0001, respectively, Fig. 9A-D). On the contrary, TNFα expression was significantly higher in DS-treated cells (14-fold, *P* < 0.001). Cytokine receptor expression showed a similar pattern. The expression of IL-6R, IL-4R, INFγR, and TNFR1 was significantly upregulated in the DS-treated group compared to those treated with NS at short term and long-term cultures (Fig. 9E-H).

## Discussion

In this work, we examined the effect of the diabetic microenvironment on the regenerative capacities of ASCs. We used diabetic serum from type 2 diabetic patients to physiologically mimic the local cellular diabetic microenvironment [28, 29]. ASCs cultured in diabetic serum suffered accelerated apoptosis and increased intracellular oxidative stress in both the short- and long-term culture. Earlier in vivo studies showed increased apoptosis, phenotypic changes, and oxidative stress in preadipocytes isolated from diabetic patients [40, 47]. The increased rate of late apoptosis of ASCs cultured in DS may be caused by the deleterious effect of oxidative stress on the cells’ membrane integrity [48], while the increased intracellular ROS may explain the higher apoptosis observed in these cells [49]. Proteomic data analysis showed upregulations of specific proteins responsible for cell apoptosis and ROS production, such as the actin cytoskeleton, which was proposed as the critical regulator of apoptosis and aging. Actin accumulation due to low turnover could trigger ROS production [50, 51]. Proteomic data also showed that disulphide isomerases were decreased in diabetic serum after 6 days and after one month, most probably due to hyperglycaemic oxidative stress [52]. Clusterin was inhibited in ASCs cultured in DS after 6 days, but further increased after one month. As clusterin acts as a protective protein against oxidative stress-related apoptosis [53–55], this increase may present a protective mechanism induced by ASCs to adapt to oxidative stress in long term culture. Clusterin is also critical for the function of the DNA repair gene, the XRCC5 encoded protein [56]. Both Clusterin and XRCC5 were decreased after 6 days of culture in DS, which may denote a possible DNA repair impairment. Our data showed that DS had significantly increased heat shock proteins 90-α (HSP 90-α) or EL52 in ASCs, one of the heat shock proteins (HSP) that regulates autophagy and necroptosis. This high expression may be mediated through receptor-interacting protein kinase 1 (RIPK1) [57], suggesting mitochondrial dysfunction, increased ROS production, and increased insulin resistance [58].

The phenotypic changes in ASCs in both short- and long-term culture in DS are in accordance with previous studies that showed an alteration in the expression of surface markers in ASCs isolated from diabetic patients [40–42]. The reduction in CD90 in MSCs reduces their immunosuppressive function and alters their osteogenic potential [43, 44]. Compromised osteogenic and adipogenic differentiation of ASCs in DS maybe related to the proinflammatory milieu and evidence for increased oxidative stress. Earlier studies showed that ROS inhibited the osteogenic potential of MSCs [59–65]. Apolipoprotein A-1 (Apo A-1) was shown to protect MSCs in the diabetic microenvironment from oxidative stress, reduce apoptosis, and support their proliferation [66]. Apo A-1 also reduced dendritic cell differentiation and compromised their proinflammatory potentials [67], increased endothelial cell proliferation, and enhanced angiogenesis [68]. MSCs isolated from apoA-1^−/−^ mice showed defective osteogenesis and bone formation [69]. Our data showed a decrease in Apo A-1in ASCs after 6 days, suggesting its possible role in defective osteogenic differentiation and angiogenesis.

Cawthorn et al. reported that TNFα inhibits adipogenic differentiation of ASCs by binding to TNF-R1 [70]. Data from our study showed significant upregulation of TNFα, TNF-R, which may account for the low adipogenic and osteogenic differentiation capacity of ASCs. TNFα treatment was previously reported to downregulate the expression of TGFβ in ASCs [71]. In our study, upregulated expression of TNFα and TNF-R1 was accompanied by downregulation of TGFβ after one month of DS treatment. Also, proteomic data showed that heat shock proteins 90-β (HSP90AB1) was decreased in ASCs cultured in DS after 6 days. Inhibition of HSP90AB1 is associated with downregulation of TGFβ, and this may explain its decreased expression after 6 days of culture in DS [72]. Inhibition of TGFβ in diabetic mice was associated with a decrease in VEGFA [73]. In our study, the decrease in TGFβ and increase in IFNγR in ASCs cultured in DS may explain the decrease in the level of VEGFA and account for the decreased angiogenesis capacity [74]. This may add to the possible mechanism of further deterioration of the angiogenic potentiality of ASCs with extended culture in DS. Overexpression of TNF-R1 and IFNγR in ASCs cultured in DS may also explain the defective angiogenesis due to augmented oxidative stress, as indicated by intracellular ROS upregulation [70, 74].

Proteomic data showed that Annexin A1 protein decreased after 1 month of culture of ASCs in DS. This was associated with a decrease in IL-6 and IL-8 production as well. Annexin A1 is a potent anti-inflammatory mediator, and its inhibition was shown to downregulate IL-6 and 8 [75]. IL-8 and IL-6 are critical for the conversion of macrophages to their M2 immune-suppressing phenotype [76–78]. IL-6 secreted from MSCs was shown to aid in the polarization of macrophages to the M2 phenotype as well [77, 78]. This may suggest an inhibition of the immunomodulatory function of ASCs in long term culture.

Our data also point to the observed resistance to oxidative stress as an adaptive mechanism of ASCs when cultured in diabetic microenvironments. Pyruvate kinase M2 (PKM2) was more expressed in ASCs in DS compared to NS after 6 days of culture. This high expression can be correlated with the activation of the protective mechanism of ASCs against the DS microenvironment, as suggested by previous findings [79], since chronic inflammation related to diabetes tends to increase PKM2 levels [28, 80]. Prolonged exposure of MSCs to oxidative stress upregulated SIRT1 expression, which activates the antioxidant protective factors that inhibit oxidative stress through AMPK activation [81–85]. In this study, upregulation of SIRT1 in ASCs was concomitant with the increased ROS level in long- and short-term cultures. In addition, our results showed an upregulation of OCT4 in ASCs cultured in DS in long-term culture. Since OCT4 is a SIRT1 inducer [86], this may indicate a mechanism by which these cells can withstand oxidative stress [82, 83]. Our data showed that TERF1 expression increased after 6 days of culture in cells cultured in DS, which may indicate some sort of ASC resistance to senescence after 6 days of culture in DS. A decrease in TERF1 is an indicator of an increase in telomere shortening [87, 88] and was reported to be elicited by acute oxidative stress and cellular senescence [89].

Our data suggest that manipulation of the culture condition of ASCs prior to transplantation in diabetic patients may be necessary to withstand the deleterious effects of the diabetic microenvironment. A recent study showed that preconditioning MSCs isolated from diabetic mice with N-acetyl cysteine (NAC) decreased their apoptosis and oxidative stress injury [90]. Transplantation of ASCs with antioxidant factors may enhance their regenerative potential in the diabetic microenvironment [15]. Since the intracellular oxidative stress of ASCs increases with their presence in the diabetic microenvironment, the readiness of the patients for cell transplantation should be determined. This is critical since the redox status of the patient is variant among diabetic patients [91, 92]. Reaching a balance between oxidants and antioxidants may enhance the regenerative potential of ASCs [91–93]. This may be achieved by prescribing antioxidant dietary supplements to patients prior to transplantation, however, their effectiveness is still under study [94]. Clinical trials showed that the oral hypoglycemic drug metformin exerts antioxidant function through upregulating antioxidants such as superoxide dismutase (SOD) and glutathione peroxidase (GPx) and thus protects against DNA damage [95–97]. The use of metformin reduces the diabetic oxidative stress may thus be helpful for diabetic patients undergoing ASC transplantation. Another important factor to be considered is the dose of transplanted cells in the harsh diabetic microenvironment [98, 99]. Meticulous clinical studies should confirm the precise dose of these cells.

## Conclusion

This study provides evidence that the diabetic microenvironment is deleterious to the regenerative capacity of ASCs. The diabetic microenvironment altered the phenotype and compromised the adipogenic and osteogenic and angiogenic differentiation of ASCs. Their immunomodulatory profile and angiogenic potential were also compromised in a time-dependent manner. Accumulated ROS resulting from oxidative stress and the inflammatory microenvironment are directly correlated with this compromised regenerative capacity. Upregulation of oxidative stress-resistant factors such as; SIRT1, TERF1, PKM2 after 6 days and SIRT1, OCT4, and Clusterin after 1 month of culture in DS may denote an adaptation mechanism to the increasing oxidative stress in the diabetic microenvironment. The deterioration of the regenerative capacity of ASCs suggests the need for optimization to the cell culture condition prior to transplantation by promoting resistance to enhanced oxidative stress.

### Limitations of the study and future directions

The mechanism by which the diabetic microenvironment deteriorates the regenerative function of ASCs requires further investigation. Of special importance is a more detailed analysis of the turning point after which the ASCs resistance to ROS deteriorates and the cells start to suffer DNA damage and cellular death. Examination of these effects in an organoid model or 3-cell culture can also shed light on the effect of diabetic serum on ASCs in a more relevant physiological model, one that is more similar to the in vivo environment and can be patient-specific. Manipulation of the culture conditions of ASCs by enhancing ROS resistance can prove direct evidence of the diabetic deleterious effect.

### Electronic supplementary material

Below is the link to the electronic supplementary material.

Supplementary Material 1

Supplementary Material 2

Supplementary Material 3

Supplementary Material 4

Supplementary Material 5

Supplementary Material 6

Supplementary Material 7

Supplementary Material 8

Supplementary Material 9

Supplementary Material 10

Supplementary Material 11

## Data Availability

All data presented in this review are totally available and present in the text. The datasets generated and/or analysed during the current study are available in the PRIDE repository, dataset link: https://www.ebi.ac.uk/pride/archive/projects/PXD036116/privatehttps://www.ebi.ac.uk/pride/archive/projects/PXD036116/private with accession number: PXD036116.

## Abbreviations

Apo A-1: Apolipoprotein A-1
ASCs: Adipose Mesenchymal Stromal Cells
DS: Diabetic Patient Serum
GPx: Glutathione Peroxidase
HSP90: Heat Shock Protein 90
HSP 90-α: Heat Shock Proteins 90-α
HSP90AB: Heat Shock Proteins 90-β
MSCs: Mesenchymal Stromal Cells
NAC: N-Acetyl Cysteine
NS: Normal Individual Serum
PKM2: Pyruvate Kinase M2
RIPK1: Receptor-Interacting Protein Kinase
1ROS: Reactive Oxygen Species
SOD: Superoxide Dismutase
