# Supplementary material for: Diabetic microenvironment deteriorates the regenerative capacities of adipose mesenchymal stromal cells
Source: Diabetol Metab Syndr. 2024 Jun 16;16:131. doi: 10.1186/s13098-024-01365-1 (PMC11181634; doi:10.1186/s13098-024-01365-1)
Supplement: Supplementary file 4 — Supplementary Material 4 [file 13098_2024_1365_MOESM4_ESM.docx]

| source | Pathway name | Term id | Adjusted p value | fold_enrichment | -log10(Adjusted p value) | Term size | Query size | Intersection size |
| --- | --- | --- | --- | --- | --- | --- | --- | --- |
| KEGG | Complement and coagulation cascades | KEGG:04610 | 2.42E-15 | 0.096386 | 14.61618 | 83 | 31 | 8 |
| KEGG | Lipid and atherosclerosis | KEGG:05417 | 3.84E-05 | 0.019139 | 4.415405 | 209 | 31 | 4 |
| KEGG | IL-17 signaling pathway | KEGG:04657 | 0.002436 | 0.021978 | 2.613397 | 91 | 31 | 2 |
| KEGG | Th17 cell differentiation | KEGG:04659 | 0.00287 | 0.019231 | 2.542118 | 104 | 31 | 2 |
| KEGG | Leukocyte transendothelial migration | KEGG:04670 | 0.00312 | 0.018182 | 2.505886 | 110 | 31 | 2 |
| KEGG | Apoptosis | KEGG:04210 | 0.003913 | 0.015385 | 2.407477 | 130 | 31 | 2 |
| KEGG | Necroptosis | KEGG:04217 | 0.004846 | 0.013158 | 2.314611 | 152 | 31 | 2 |
| KEGG | PI3K-Akt signaling pathway | KEGG:04151 | 0.018986 | 0.005848 | 1.721571 | 342 | 31 | 2 |
| KEGG | Type II diabetes mellitus | KEGG:04930 | 0.031891 | 0.022222 | 1.496331 | 45 | 31 | 1 |
| KEGG | Glycolysis / Gluconeogenesis | KEGG:00010 | 0.041506 | 0.015625 | 1.381886 | 64 | 31 | 1 |
| KEGG | PPAR signaling pathway | KEGG:03320 | 0.044303 | 0.013514 | 1.353572 | 74 | 31 | 1 |
| REAC | Immune System | REAC:R-HSA-168256 | 1.89E-17 | 0.008819 | 16.72354 | 2041 | 31 | 18 |
| REAC | Innate Immune System | REAC:R-HSA-168249 | 3.05E-15 | 0.012797 | 14.5157 | 1094 | 31 | 14 |
| REAC | Cellular responses to stress | REAC:R-HSA-2262752 | 3.66E-08 | 0.010652 | 7.436519 | 751 | 31 | 8 |
| REAC | Cellular responses to stimuli | REAC:R-HSA-8953897 | 4.07E-08 | 0.010458 | 7.390406 | 765 | 31 | 8 |
| REAC | Activation of C3 and C5 | REAC:R-HSA-174577 | 4.68E-08 | 0.428571 | 7.329754 | 7 | 31 | 3 |
| REAC | Signal Transduction | REAC:R-HSA-162582 | 2.16E-07 | 0.00436 | 6.665546 | 2523 | 31 | 11 |
| REAC | Regulation of Complement cascade | REAC:R-HSA-977606 | 1.86E-06 | 0.039604 | 5.731104 | 101 | 31 | 4 |
| REAC | Autophagy | REAC:R-HSA-9612973 | 7.53E-06 | 0.026846 | 5.123206 | 149 | 31 | 4 |
| REAC | Cellular response to chemical stress | REAC:R-HSA-9711123 | 8.84E-06 | 0.025478 | 5.053353 | 157 | 31 | 4 |
| REAC | Signaling by Rho GTPases | REAC:R-HSA-194315 | 8.87E-06 | 0.008547 | 5.052198 | 702 | 31 | 6 |
| REAC | Signaling by Rho GTPases, Miro GTPases and RHOBTB3 | REAC:R-HSA-9716542 | 9.80E-06 | 0.008368 | 5.008952 | 717 | 31 | 6 |
| REAC | RHO GTPase cycle | REAC:R-HSA-9012999 | 1.74E-05 | 0.011136 | 4.760615 | 449 | 31 | 5 |
| REAC | Signaling by Interleukins | REAC:R-HSA-449147 | 1.92E-05 | 0.01087 | 4.717629 | 460 | 31 | 5 |
| REAC | Initial triggering of complement | REAC:R-HSA-166663 | 4.07E-05 | 0.039474 | 4.390725 | 76 | 31 | 3 |
| REAC | Signaling by VEGF | REAC:R-HSA-194138 | 7.96E-05 | 0.028846 | 4.099271 | 104 | 31 | 3 |
| REAC | Interleukin-4 and Interleukin-13 signaling | REAC:R-HSA-6785807 | 9.07E-05 | 0.027273 | 4.042376 | 110 | 31 | 3 |
| REAC | Cytokine Signaling in Immune system | REAC:R-HSA-1280215 | 9.62E-05 | 0.007092 | 4.016634 | 705 | 31 | 5 |
| REAC | Cell-extracellular matrix interactions | REAC:R-HSA-446353 | 0.000134 | 0.111111 | 3.872089 | 18 | 31 | 2 |
| REAC | Apoptotic cleavage of cellular proteins | REAC:R-HSA-111465 | 0.000477 | 0.054054 | 3.321687 | 37 | 31 | 2 |
| REAC | MAP2K and MAPK activation | REAC:R-HSA-5674135 | 0.00052 | 0.051282 | 3.283733 | 39 | 31 | 2 |
| REAC | Interleukin-12 signaling | REAC:R-HSA-9020591 | 0.000687 | 0.042553 | 3.16294 | 47 | 31 | 2 |
| REAC | Apoptotic execution phase | REAC:R-HSA-75153 | 0.000777 | 0.039216 | 3.109676 | 51 | 31 | 2 |
| REAC | Interleukin-12 family signaling | REAC:R-HSA-447115 | 0.000955 | 0.035088 | 3.01992 | 57 | 31 | 2 |
| REAC | Selective autophagy | REAC:R-HSA-9663891 | 0.001752 | 0.025 | 2.756364 | 80 | 31 | 2 |
| REAC | Cellular response to heat stress | REAC:R-HSA-3371556 | 0.002025 | 0.022989 | 2.693563 | 87 | 31 | 2 |
| REAC | Cell-Cell communication | REAC:R-HSA-1500931 | 0.003787 | 0.015748 | 2.421711 | 127 | 31 | 2 |
| REAC | Macroautophagy | REAC:R-HSA-1632852 | 0.003928 | 0.014925 | 2.405861 | 134 | 31 | 2 |
| REAC | eNOS activation | REAC:R-HSA-203615 | 0.009083 | 0.090909 | 2.041765 | 11 | 31 | 1 |
| REAC | Metabolism of nitric oxide: NOS3 activation and regulation | REAC:R-HSA-202131 | 0.011874 | 0.066667 | 1.925401 | 15 | 31 | 1 |
| REAC | The NLRP3 inflammasome | REAC:R-HSA-844456 | 0.012351 | 0.0625 | 1.908309 | 16 | 31 | 1 |
| REAC | MAPK1/MAPK3 signaling | REAC:R-HSA-5684996 | 0.012469 | 0.007435 | 1.904178 | 269 | 31 | 2 |
| REAC | Extracellular matrix organization | REAC:R-HSA-1474244 | 0.014597 | 0.006711 | 1.835727 | 298 | 31 | 2 |
| REAC | MAPK family signaling cascades | REAC:R-HSA-5683057 | 0.015259 | 0.006494 | 1.816483 | 308 | 31 | 2 |
| REAC | Regulation of TLR by endogenous ligand | REAC:R-HSA-5686938 | 0.015259 | 0.047619 | 1.816483 | 21 | 31 | 1 |
| REAC | Inflammasomes | REAC:R-HSA-622312 | 0.015259 | 0.047619 | 1.816483 | 21 | 31 | 1 |
| REAC | VEGFR2 mediated vascular permeability | REAC:R-HSA-5218920 | 0.018837 | 0.037037 | 1.724981 | 27 | 31 | 1 |
| REAC | RIPK1-mediated regulated necrosis | REAC:R-HSA-5213460 | 0.019188 | 0.035714 | 1.716979 | 28 | 31 | 1 |
| REAC | Regulation of necroptotic cell death | REAC:R-HSA-5675482 | 0.019188 | 0.035714 | 1.716979 | 28 | 31 | 1 |
| REAC | Detoxification of Reactive Oxygen Species | REAC:R-HSA-3299685 | 0.02343 | 0.028571 | 1.63023 | 35 | 31 | 1 |
| REAC | Cell Cycle, Mitotic | REAC:R-HSA-69278 | 0.039104 | 0.00365 | 1.407784 | 548 | 31 | 2 |
| WP | Complement system | WP:WP2806 | 1.20E-08 | 0.055556 | 7.920819 | 90 | 31 | 5 |
| WP | VEGFA-VEGFR2 signaling pathway | WP:WP3888 | 5.13E-07 | 0.014458 | 6.289883 | 415 | 31 | 6 |
| WP | Complement activation | WP:WP545 | 1.14E-06 | 0.142857 | 5.941669 | 21 | 31 | 3 |
| WP | Fas ligand pathway and stress induction of heat shock proteins | WP:WP314 | 0.000856 | 0.045455 | 3.067275 | 44 | 31 | 2 |
| WP | Apoptosis-related network due to altered Notch3 in ovarian cancer | WP:WP2864 | 0.001074 | 0.038462 | 2.969138 | 52 | 31 | 2 |
| WP | PPAR-alpha pathway | WP:WP2878 | 0.020953 | 0.04 | 1.678756 | 25 | 31 | 1 |
| WP | Th17 cell differentiation pathway | WP:WP5130 | 0.039041 | 0.014493 | 1.408485 | 69 | 31 | 1 |
| WP | T-cell receptor signaling pathway | WP:WP69 | 0.047323 | 0.011111 | 1.324926 | 90 | 31 | 1 |
| WP | TNF-alpha signaling pathway | WP:WP231 | 0.048334 | 0.010753 | 1.315748 | 93 | 31 | 1 |
