# Supplementary material for: Diabetic microenvironment deteriorates the regenerative capacities of adipose mesenchymal stromal cells
Source: Diabetol Metab Syndr. 2024 Jun 16;16:131. doi: 10.1186/s13098-024-01365-1 (PMC11181634; doi:10.1186/s13098-024-01365-1)
Supplement: Supplementary file 5 — Supplementary Material 5 [file 13098_2024_1365_MOESM5_ESM.docx]

| source | Pathway name | Term id | Adjusted p value | Fold enrichment | -log10(Adjusted p value) | Term size | Query size | Intersection size |
| --- | --- | --- | --- | --- | --- | --- | --- | --- |
| KEGG | Complement and coagulation cascades | KEGG:04610 | 1.23E-17 | 0.108434 | 16.91009 | 83 | 31 | 9 |
| REAC | Regulation of IGF transport and uptake by IGFBPs | REAC:R-HSA-381426 | 2.11E-25 | 0.104839 | 24.67572 | 124 | 31 | 13 |
| REAC | Innate Immune System | REAC:R-HSA-168249 | 3.29E-17 | 0.013711 | 16.4828 | 1094 | 31 | 15 |
| REAC | Regulation of Complement cascade | REAC:R-HSA-977606 | 1.47E-12 | 0.069307 | 11.83268 | 101 | 31 | 7 |
| REAC | Complement cascade | REAC:R-HSA-166658 | 2.64E-12 | 0.063063 | 11.5784 | 111 | 31 | 7 |
| REAC | Cellular responses to stress | REAC:R-HSA-2262752 | 2.25E-08 | 0.010652 | 7.647817 | 751 | 31 | 8 |
| REAC | Cellular responses to stimuli | REAC:R-HSA-8953897 | 2.51E-08 | 0.010458 | 7.600326 | 765 | 31 | 8 |
| REAC | Chaperone Mediated Autophagy | REAC:R-HSA-9613829 | 0.000205 | 0.090909 | 3.687328 | 22 | 31 | 2 |
| REAC | Autophagy | REAC:R-HSA-9612973 | 0.000213 | 0.020134 | 3.670747 | 149 | 31 | 3 |
| REAC | Signaling by Interleukins | REAC:R-HSA-449147 | 0.000287 | 0.008696 | 3.541601 | 460 | 31 | 4 |
| REAC | Cytokine Signaling in Immune system | REAC:R-HSA-1280215 | 0.001355 | 0.005674 | 2.867914 | 705 | 31 | 4 |
| REAC | Interleukin-4 and Interleukin-13 signaling | REAC:R-HSA-6785807 | 0.004005 | 0.018182 | 2.397443 | 110 | 31 | 2 |
| REAC | Alternative complement activation | REAC:R-HSA-173736 | 0.006352 | 0.2 | 2.197083 | 5 | 31 | 1 |
| REAC | Toll-like Receptor Cascades | REAC:R-HSA-168898 | 0.00689 | 0.012987 | 2.161801 | 154 | 31 | 2 |
| REAC | Cell recruitment (pro-inflammatory response) | REAC:R-HSA-9664424 | 0.021908 | 0.038462 | 1.659393 | 26 | 31 | 1 |
| REAC | Detoxification of Reactive Oxygen Species | REAC:R-HSA-3299685 | 0.027746 | 0.028571 | 1.556804 | 35 | 31 | 1 |
| REAC | Interleukin-12 signaling | REAC:R-HSA-9020591 | 0.035142 | 0.021277 | 1.454176 | 47 | 31 | 1 |
| REAC | Interleukin-12 family signaling | REAC:R-HSA-447115 | 0.041126 | 0.017544 | 1.385884 | 57 | 31 | 1 |
| WP | Complement system | WP:WP2806 | 1.08E-10 | 0.066667 | 9.966576 | 90 | 31 | 6 |
| WP | VEGFA-VEGFR2 signaling pathway | WP:WP3888 | 1.16E-05 | 0.012048 | 4.934715 | 415 | 31 | 5 |
| WP | Apoptosis-related network due to altered Notch3 in ovarian cancer | WP:WP2864 | 1.36E-05 | 0.057692 | 4.866161 | 52 | 31 | 3 |
| WP | Glycolysis in senescence | WP:WP5049 | 5.46E-05 | 0.181818 | 4.262644 | 11 | 31 | 2 |
| WP | Interleukin-1 induced activation of NF-kB | WP:WP3656 | 0.010185 | 0.1 | 1.992027 | 10 | 31 | 1 |
| WP | PPAR-alpha pathway | WP:WP2878 | 0.019147 | 0.04 | 1.717891 | 25 | 31 | 1 |
| WP | IL1 and megakaryocytes in obesity | WP:WP2865 | 0.019147 | 0.04 | 1.717891 | 25 | 31 | 1 |
| WP | Oxidative damage response | WP:WP3941 | 0.027893 | 0.025 | 1.554509 | 40 | 31 | 1 |
| WP | IL-1 signaling pathway | WP:WP195 | 0.034549 | 0.018519 | 1.461566 | 54 | 31 | 1 |
| WP | PPAR signaling pathway | WP:WP3942 | 0.04018 | 0.014925 | 1.395991 | 67 | 31 | 1 |
