# Supplementary material for: Diabetic microenvironment deteriorates the regenerative capacities of adipose mesenchymal stromal cells
Source: Diabetol Metab Syndr. 2024 Jun 16;16:131. doi: 10.1186/s13098-024-01365-1 (PMC11181634; doi:10.1186/s13098-024-01365-1)
Supplement: Supplementary file 6 — Supplementary Material 6 [file 13098_2024_1365_MOESM6_ESM.docx]

| source | Pathway Name | Term id | Adjusted p value | Fold enrichment | -log10(Adjusted p value) | Term size | Query size | Intersection size |
| --- | --- | --- | --- | --- | --- | --- | --- | --- |
| KEGG | Complement and coagulation cascades | KEGG:04610 | 4.04E-09 | 0.060241 | 8.393619 | 83 | 21 | 5 |
| REAC | Immune System | REAC:R-HSA-168256 | 1.90E-08 | 0.0049 | 7.721246 | 2041 | 21 | 10 |
| REAC | Regulation of IGF transport and uptake by IGFBPs | REAC:R-HSA-381426 | 2.01E-08 | 0.040323 | 7.696804 | 124 | 21 | 5 |
| REAC | Innate Immune System | REAC:R-HSA-168249 | 3.85E-08 | 0.007313 | 7.414539 | 1094 | 21 | 8 |
| REAC | Neutrophil degranulation | REAC:R-HSA-6798695 | 0.003619 | 0.006303 | 2.441357 | 476 | 21 | 3 |
| REAC | Autophagy | REAC:R-HSA-9612973 | 0.006449 | 0.013423 | 2.1905 | 149 | 21 | 2 |
| REAC | Apoptotic cleavage of cellular proteins | REAC:R-HSA-111465 | 0.029526 | 0.027027 | 1.529794 | 37 | 21 | 1 |
| REAC | DNA Damage Recognition in GG-NER | REAC:R-HSA-5696394 | 0.029526 | 0.026316 | 1.529794 | 38 | 21 | 1 |
| REAC | MAP2K and MAPK activation | REAC:R-HSA-5674135 | 0.029938 | 0.025641 | 1.523784 | 39 | 21 | 1 |
| REAC | Apoptotic execution phase | REAC:R-HSA-75153 | 0.031865 | 0.019608 | 1.496683 | 51 | 21 | 1 |
| REAC | VEGFA-VEGFR2 Pathway | REAC:R-HSA-4420097 | 0.045858 | 0.010638 | 1.338587 | 94 | 21 | 1 |
| REAC | RUNX1 regulates genes involved in megakaryocyte differentiation | REAC:R-HSA-8936459 | 0.046239 | 0.010309 | 1.334987 | 97 | 21 | 1 |
| REAC | Signaling by VEGF | REAC:R-HSA-194138 | 0.048436 | 0.009615 | 1.314834 | 104 | 21 | 1 |
| REAC | Nucleotide Excision Repair | REAC:R-HSA-5696398 | 0.049994 | 0.009174 | 1.301086 | 109 | 21 | 1 |
| WP | Complement system | WP:WP2806 | 0.002144 | 0.022222 | 2.66873 | 90 | 21 | 2 |
| WP | TGF-beta signaling in thyroid cells for epithelial-mesenchymal transition | WP:WP3859 | 0.01655 | 0.055556 | 1.781206 | 18 | 21 | 1 |
| WP | PPAR-alpha pathway | WP:WP2878 | 0.020873 | 0.04 | 1.680425 | 25 | 21 | 1 |
| WP | IL1 and megakaryocytes in obesity | WP:WP2865 | 0.020873 | 0.04 | 1.680425 | 25 | 21 | 1 |
| WP | Oxidative damage response | WP:WP3941 | 0.027749 | 0.025 | 1.556757 | 40 | 21 | 1 |
| WP | PPAR signaling pathway | WP:WP3942 | 0.033035 | 0.014925 | 1.48103 | 67 | 21 | 1 |
| WP | T-cell receptor signaling pathway | WP:WP69 | 0.039897 | 0.011111 | 1.399063 | 90 | 21 | 1 |
