# Supplementary material for: Diabetic microenvironment deteriorates the regenerative capacities of adipose mesenchymal stromal cells
Source: Diabetol Metab Syndr. 2024 Jun 16;16:131. doi: 10.1186/s13098-024-01365-1 (PMC11181634; doi:10.1186/s13098-024-01365-1)
Supplement: Supplementary file 7 — Supplementary Material 7 [file 13098_2024_1365_MOESM7_ESM.docx]

| GO | Term id | Adjusted p value | -log10(Adjusted p value) | Term size | Query size | Freq |
| --- | --- | --- | --- | --- | --- | --- |
| peroxidase activity | GO:0004601 | 0.029592 | 1.528821 | 52 | 21 | 1 |
| oxidoreductase activity | GO:0016491 | 0.039781 | 1.400321 | 755 | 21 | 2 |
| antioxidant activity | GO:0016209 | 0.040112 | 1.396724 | 84 | 21 | 1 |
| response to stress | GO:0006950 | 4.53E-13 | 12.34379 | 3851 | 21 | 16 |
| innate immune response | GO:0045087 | 1.25E-08 | 7.901709 | 907 | 21 | 8 |
| regulation of response to stress | GO:0080134 | 2.55E-06 | 5.592719 | 1312 | 21 | 7 |
| astrocyte differentiation | GO:0048708 | 0.00219 | 2.659572 | 76 | 21 | 2 |
| cell differentiation | GO:0030154 | 0.002245 | 2.648806 | 4160 | 21 | 7 |
| antimicrobial humoral immune response mediated by antimicrobial peptide | GO:0061844 | 0.002388 | 2.622037 | 82 | 21 | 2 |
| complement activation, classical pathway | GO:0006958 | 0.003601 | 2.443624 | 107 | 21 | 2 |
| response to interferon-gamma | GO:0034341 | 0.006103 | 2.214439 | 149 | 21 | 2 |
| regulation of defense response | GO:0031347 | 0.006257 | 2.203608 | 629 | 21 | 3 |
| tumor necrosis factor production | GO:0032640 | 0.006571 | 2.182388 | 160 | 21 | 2 |
| regulation of tumor necrosis factor production | GO:0032680 | 0.006571 | 2.182388 | 160 | 21 | 2 |
| positive regulation of interferon-gamma-mediated signaling pathway | GO:0060335 | 0.008706 | 2.060161 | 6 | 21 | 1 |
| positive regulation of response to interferon-gamma | GO:0060332 | 0.008706 | 2.060161 | 6 | 21 | 1 |
| reactive oxygen species metabolic process | GO:0072593 | 0.01057 | 1.975942 | 221 | 21 | 2 |
| positive regulation of apoptotic cell clearance | GO:2000427 | 0.010895 | 1.962768 | 8 | 21 | 1 |
| regulation of innate immune response | GO:0045088 | 0.012212 | 1.913202 | 245 | 21 | 2 |
| positive regulation of macrophage differentiation | GO:0045651 | 0.017187 | 1.7648 | 16 | 21 | 1 |
| regulation of interferon-gamma-mediated signaling pathway | GO:0060334 | 0.017187 | 1.7648 | 16 | 21 | 1 |
| negative regulation of megakaryocyte differentiation | GO:0045653 | 0.018641 | 1.729539 | 18 | 21 | 1 |
| negative regulation of humoral immune response | GO:0002921 | 0.018641 | 1.729539 | 18 | 21 | 1 |
| cellular response to peptide | GO:1901653 | 0.018993 | 1.721404 | 349 | 21 | 2 |
| negative regulation of bone mineralization | GO:0030502 | 0.019355 | 1.71321 | 19 | 21 | 1 |
| negative regulation of glial cell differentiation | GO:0045686 | 0.02348 | 1.629299 | 25 | 21 | 1 |
| cell differentiation involved in embryonic placenta development | GO:0060706 | 0.025523 | 1.593067 | 28 | 21 | 1 |
| regulation of astrocyte differentiation | GO:0048710 | 0.025523 | 1.593067 | 28 | 21 | 1 |
| regulation of vascular endothelial growth factor receptor signaling pathway | GO:0030947 | 0.025929 | 1.58621 | 29 | 21 | 1 |
| interferon-gamma-mediated signaling pathway | GO:0060333 | 0.030018 | 1.522625 | 36 | 21 | 1 |
| negative regulation of interleukin-6 production | GO:0032715 | 0.035324 | 1.451932 | 46 | 21 | 1 |
| apoptotic cell clearance | GO:0043277 | 0.038708 | 1.412205 | 52 | 21 | 1 |
| hydrogen peroxide metabolic process | GO:0042743 | 0.039135 | 1.407429 | 53 | 21 | 1 |
| endoderm formation | GO:0001706 | 0.039971 | 1.398256 | 55 | 21 | 1 |
| vascular endothelial growth factor receptor signaling pathway | GO:0048010 | 0.039971 | 1.398256 | 55 | 21 | 1 |
| regulation of cytokine production involved in inflammatory response | GO:1900015 | 0.040534 | 1.392178 | 56 | 21 | 1 |
| megakaryocyte differentiation | GO:0030219 | 0.042197 | 1.374717 | 59 | 21 | 1 |
| positive regulation of interleukin-1 beta production | GO:0032731 | 0.042581 | 1.370785 | 60 | 21 | 1 |
| positive regulation of reactive oxygen species metabolic process | GO:2000379 | 0.046188 | 1.335474 | 68 | 21 | 1 |
| nitric oxide biosynthetic process | GO:0006809 | 0.046522 | 1.33234 | 69 | 21 | 1 |
| extrinsic apoptotic signaling pathway in absence of ligand | GO:0097192 | 0.046522 | 1.33234 | 69 | 21 | 1 |
| negative regulation of innate immune response | GO:0045824 | 0.047664 | 1.321809 | 72 | 21 | 1 |
| nitric oxide metabolic process | GO:0046209 | 0.049105 | 1.308872 | 75 | 21 | 1 |
