# Supplementary material for: Diabetic microenvironment deteriorates the regenerative capacities of adipose mesenchymal stromal cells
Source: Diabetol Metab Syndr. 2024 Jun 16;16:131. doi: 10.1186/s13098-024-01365-1 (PMC11181634; doi:10.1186/s13098-024-01365-1)
Supplement: Supplementary file 8 — Supplementary Material 8 [file 13098_2024_1365_MOESM8_ESM.docx]

| GO | Term id | Adjusted p value | -log10(Adjusted p value) | Term size | Query size | Freq |
| --- | --- | --- | --- | --- | --- | --- |
| antioxidant activity | GO:0016209 | 1.03E-06 | 5.98678 | 84 | 31 | 4 |
| nitric-oxide synthase regulator activity | GO:0030235 | 3.02E-05 | 4.52023 | 7 | 31 | 2 |
| peroxidase activity | GO:0004601 | 0.001127 | 2.947895 | 52 | 31 | 2 |
| pyruvate kinase activity | GO:0004743 | 0.002598 | 2.585295 | 2 | 31 | 1 |
| interleukin-8 binding | GO:0019959 | 0.003599 | 2.443766 | 3 | 31 | 1 |
| tumor necrosis factor binding | GO:0043120 | 0.005401 | 2.267518 | 5 | 31 | 1 |
| nitric-oxide synthase binding | GO:0050998 | 0.0102 | 1.991393 | 13 | 31 | 1 |
| innate immune response | GO:0045087 | 1.87E-14 | 13.72866 | 907 | 31 | 13 |
| regulation of cellular protein metabolic process | GO:0032268 | 2.56E-13 | 12.59173 | 2370 | 31 | 16 |
| immune response | GO:0006955 | 2.65E-13 | 12.57668 | 1906 | 31 | 15 |
| regulation of cellular metabolic process | GO:0031323 | 5.55E-13 | 12.25548 | 6004 | 31 | 21 |
| immune system process | GO:0002376 | 2.10E-12 | 11.67741 | 2781 | 31 | 16 |
| regulation of nitrogen compound metabolic process | GO:0051171 | 2.50E-12 | 11.60214 | 5654 | 31 | 20 |
| cell differentiation | GO:0030154 | 5.32E-10 | 9.274007 | 4160 | 31 | 16 |
| cellular developmental process | GO:0048869 | 5.66E-10 | 9.246849 | 4184 | 31 | 16 |
| regulation of response to stress | GO:0080134 | 5.66E-10 | 9.246849 | 1312 | 31 | 11 |
| cell death | GO:0008219 | 3.23E-09 | 8.491382 | 2074 | 31 | 12 |
| regulation of cell death | GO:0010941 | 3.48E-09 | 8.457862 | 1592 | 31 | 11 |
| B cell mediated immunity | GO:0019724 | 1.30E-08 | 7.887065 | 200 | 31 | 6 |
| positive regulation of nitric oxide biosynthetic process | GO:0045429 | 6.24E-08 | 7.204533 | 39 | 31 | 4 |
| positive regulation of nitric oxide metabolic process | GO:1904407 | 7.53E-08 | 7.123453 | 41 | 31 | 4 |
| regulation of apoptotic cell clearance | GO:2000425 | 1.82E-07 | 6.740267 | 10 | 31 | 3 |
| regulation of nitric oxide biosynthetic process | GO:0045428 | 2.18E-07 | 6.661377 | 55 | 31 | 4 |
| programmed cell death | GO:0012501 | 2.45E-07 | 6.610036 | 1918 | 31 | 10 |
| regulation of nitric oxide metabolic process | GO:0080164 | 2.57E-07 | 6.589421 | 58 | 31 | 4 |
| regulation of apoptotic process | GO:0042981 | 2.69E-07 | 6.570901 | 1419 | 31 | 9 |
| nitric oxide biosynthetic process | GO:0006809 | 4.70E-07 | 6.327976 | 69 | 31 | 4 |
| positive regulation of phagocytosis | GO:0050766 | 4.93E-07 | 6.306917 | 70 | 31 | 4 |
| nitric oxide metabolic process | GO:0046209 | 6.37E-07 | 6.195934 | 75 | 31 | 4 |
| reactive nitrogen species metabolic process | GO:2001057 | 6.56E-07 | 6.183021 | 76 | 31 | 4 |
| cellular oxidant detoxification | GO:0098869 | 1.52E-06 | 5.817192 | 95 | 31 | 4 |
| regulation of phagocytosis | GO:0050764 | 1.78E-06 | 5.748881 | 99 | 31 | 4 |
| cellular response to toxic substance | GO:0097237 | 3.41E-06 | 5.466845 | 118 | 31 | 4 |
| regulation of wound healing | GO:0061041 | 4.67E-06 | 5.330622 | 128 | 31 | 4 |
| nitric oxide transport | GO:0030185 | 1.72E-05 | 4.765372 | 5 | 31 | 2 |
| response to reactive oxygen species | GO:0000302 | 1.91E-05 | 4.719244 | 193 | 31 | 4 |
| negative regulation of apoptotic process | GO:0043066 | 2.79E-05 | 4.554182 | 869 | 31 | 6 |
| negative regulation of programmed cell death | GO:0043069 | 3.11E-05 | 4.507399 | 887 | 31 | 6 |
| response to toxic substance | GO:0009636 | 3.61E-05 | 4.442536 | 231 | 31 | 4 |
| cell-cell adhesion | GO:0098609 | 3.61E-05 | 4.442536 | 913 | 31 | 6 |
| regulation of cell motility | GO:2000145 | 4.46E-05 | 4.350942 | 952 | 31 | 6 |
| tissue migration | GO:0090130 | 8.83E-05 | 4.053993 | 302 | 31 | 4 |
| cellular response to stress | GO:0033554 | 0.000165 | 3.783256 | 1875 | 31 | 7 |
| response to interferon-gamma | GO:0034341 | 0.000269 | 3.569835 | 149 | 31 | 3 |
| regulation of cell migration | GO:0030334 | 0.000339 | 3.470315 | 893 | 31 | 5 |
| regulation of cell differentiation | GO:0045595 | 0.000446 | 3.351021 | 1550 | 31 | 6 |
| positive regulation of telomerase activity | GO:0051973 | 0.00054 | 3.267522 | 34 | 31 | 2 |
| endothelial cell migration | GO:0043542 | 0.000679 | 3.168221 | 215 | 31 | 3 |
| regulation of telomerase activity | GO:0051972 | 0.001069 | 2.971178 | 50 | 31 | 2 |
| positive regulation of cell morphogenesis involved in differentiation | GO:0010770 | 0.002355 | 2.628098 | 80 | 31 | 2 |
| positive regulation of lymphocyte activation | GO:0051251 | 0.002585 | 2.587544 | 368 | 31 | 3 |
| osteoclast differentiation | GO:0030316 | 0.00348 | 2.458388 | 101 | 31 | 2 |
| telomere maintenance | GO:0000723 | 0.006414 | 2.192876 | 148 | 31 | 2 |
| endothelial cell proliferation | GO:0001935 | 0.006742 | 2.171217 | 152 | 31 | 2 |
| leukocyte differentiation | GO:0002521 | 0.007066 | 2.150856 | 563 | 31 | 3 |
| positive regulation of angiogenesis | GO:0045766 | 0.007356 | 2.133351 | 162 | 31 | 2 |
| regulation of intrinsic apoptotic signaling pathway | GO:2001242 | 0.007671 | 2.115135 | 166 | 31 | 2 |
| regulation of endothelial cell migration | GO:0010594 | 0.007753 | 2.110535 | 167 | 31 | 2 |
| regulation of leukocyte activation | GO:0002694 | 0.008701 | 2.060422 | 620 | 31 | 3 |
| regulation of adaptive immune response | GO:0002819 | 0.008991 | 2.046211 | 183 | 31 | 2 |
| negative regulation of immune response | GO:0050777 | 0.008991 | 2.046211 | 183 | 31 | 2 |
| regulation of cellular response to stress | GO:0080135 | 0.009896 | 2.004553 | 657 | 31 | 3 |
| regulation of B cell activation | GO:0050864 | 0.010032 | 1.998604 | 198 | 31 | 2 |
| epithelial cell differentiation | GO:0030855 | 0.010175 | 1.992482 | 670 | 31 | 3 |
| reactive oxygen species metabolic process | GO:0072593 | 0.011793 | 1.928384 | 221 | 31 | 2 |
| response to tumor necrosis factor | GO:0034612 | 0.015412 | 1.812148 | 262 | 31 | 2 |
| cellular response to oxidative stress | GO:0034599 | 0.016551 | 1.781179 | 274 | 31 | 2 |
| intrinsic apoptotic signaling pathway | GO:0097193 | 0.01854 | 1.73189 | 296 | 31 | 2 |
| positive regulation of defense response | GO:0031349 | 0.019195 | 1.716809 | 302 | 31 | 2 |
| ERK1 and ERK2 cascade | GO:0070371 | 0.020853 | 1.680831 | 320 | 31 | 2 |
| regulation of apoptotic signaling pathway | GO:2001233 | 0.024412 | 1.612402 | 358 | 31 | 2 |
| epithelial cell proliferation | GO:0050673 | 0.028742 | 1.541489 | 402 | 31 | 2 |
| autophagy | GO:0006914 | 0.043853 | 1.357998 | 541 | 31 | 2 |
| apoptotic signaling pathway | GO:0097190 | 0.049513 | 1.305278 | 592 | 31 | 2 |
