# Supplementary material for: Diabetic microenvironment deteriorates the regenerative capacities of adipose mesenchymal stromal cells
Source: Diabetol Metab Syndr. 2024 Jun 16;16:131. doi: 10.1186/s13098-024-01365-1 (PMC11181634; doi:10.1186/s13098-024-01365-1)
Supplement: Supplementary file 9 — Supplementary Material 9 [file 13098_2024_1365_MOESM9_ESM.docx]

| GO | Term id | Adjusted p value | -log10(Adjusted p value) | Term size | Query size | Freq. |
| --- | --- | --- | --- | --- | --- | --- |
| antioxidant activity | GO:0016209 | 6.64E-05 | 4.177719 | 84 | 31 | 3 |
| oxidoreductase activity | GO:0016491 | 0.00196 | 2.707818 | 755 | 31 | 4 |
| peroxidase activity | GO:0004601 | 0.041595 | 1.380964 | 52 | 31 | 1 |
| response to stress | GO:0006950 | 1.80E-18 | 17.74564 | 3851 | 31 | 23 |
| proteolysis | GO:0006508 | 1.51E-14 | 13.82191 | 1787 | 31 | 16 |
| wound healing | GO:0042060 | 8.16E-13 | 12.08831 | 429 | 31 | 10 |
| inflammatory response | GO:0006954 | 4.47E-12 | 11.34984 | 778 | 31 | 11 |
| cellular metabolic process | GO:0044237 | 7.59E-10 | 9.119868 | 12865 | 31 | 24 |
| cell death | GO:0008219 | 3.83E-09 | 8.417093 | 2074 | 31 | 12 |
| regulation of cell death | GO:0010941 | 4.12E-09 | 8.38489 | 1592 | 31 | 11 |
| humoral immune response | GO:0006959 | 4.89E-09 | 8.310894 | 318 | 31 | 7 |
| regulation of apoptotic process | GO:0042981 | 2.16E-08 | 7.666405 | 1419 | 31 | 10 |
| programmed cell death | GO:0012501 | 2.27E-08 | 7.644102 | 1918 | 31 | 11 |
| regulation of programmed cell death | GO:0043067 | 2.47E-08 | 7.606836 | 1448 | 31 | 10 |
| defense response to other organism | GO:0098542 | 6.60E-08 | 7.18074 | 1167 | 31 | 9 |
| regulation of apoptotic cell clearance | GO:2000425 | 1.83E-07 | 6.738029 | 10 | 31 | 3 |
| apoptotic cell clearance | GO:0043277 | 1.83E-07 | 6.737056 | 52 | 31 | 4 |
| apoptotic process | GO:0006915 | 1.99E-07 | 6.700754 | 1865 | 31 | 10 |
| lymphocyte mediated immunity | GO:0002449 | 2.32E-07 | 6.633678 | 345 | 31 | 6 |
| adaptive immune response | GO:0002250 | 6.31E-07 | 6.199718 | 718 | 31 | 7 |
| negative regulation of apoptotic process | GO:0043066 | 2.03E-06 | 5.693128 | 869 | 31 | 7 |
| acute inflammatory response | GO:0002526 | 3.12E-06 | 5.505428 | 115 | 31 | 4 |
| cell differentiation | GO:0030154 | 3.33E-06 | 5.477547 | 4160 | 31 | 12 |
| regulation of cell morphogenesis | GO:0022604 | 3.98E-06 | 5.399773 | 307 | 31 | 5 |
| negative regulation of cell death | GO:0060548 | 4.23E-06 | 5.373821 | 983 | 31 | 7 |
| cell morphogenesis | GO:0000902 | 5.04E-06 | 5.297402 | 1012 | 31 | 7 |
| positive regulation of cell differentiation | GO:0045597 | 2.55E-05 | 4.592653 | 840 | 31 | 6 |
| endothelial cell migration | GO:0043542 | 3.01E-05 | 4.521977 | 215 | 31 | 4 |
| nitric oxide biosynthetic process | GO:0006809 | 4.07E-05 | 4.390472 | 69 | 31 | 3 |
| positive regulation of response to wounding | GO:1903036 | 4.22E-05 | 4.374756 | 70 | 31 | 3 |
| reactive nitrogen species metabolic process | GO:2001057 | 5.26E-05 | 4.279404 | 76 | 31 | 3 |
| positive regulation of signal transduction | GO:0009967 | 5.55E-05 | 4.255431 | 1513 | 31 | 7 |
| response to endoplasmic reticulum stress | GO:0034976 | 5.72E-05 | 4.24289 | 258 | 31 | 4 |
| regulation of cell differentiation | GO:0045595 | 6.41E-05 | 4.193452 | 1550 | 31 | 7 |
| epithelial cell migration | GO:0010631 | 8.75E-05 | 4.058205 | 293 | 31 | 4 |
| cell motility | GO:0048870 | 9.06E-05 | 4.042864 | 1655 | 31 | 7 |
| cellular oxidant detoxification | GO:0098869 | 9.33E-05 | 4.030218 | 95 | 31 | 3 |
| tissue migration | GO:0090130 | 9.55E-05 | 4.020125 | 302 | 31 | 4 |
| cellular detoxification | GO:1990748 | 0.000135 | 3.870327 | 110 | 31 | 3 |
| cellular response to stress | GO:0033554 | 0.000177 | 3.753223 | 1875 | 31 | 7 |
| cell population proliferation | GO:0008283 | 0.000222 | 3.652946 | 1958 | 31 | 7 |
| detoxification | GO:0098754 | 0.000223 | 3.651516 | 135 | 31 | 3 |
| response to oxidative stress | GO:0006979 | 0.000282 | 3.549619 | 420 | 31 | 4 |
| negative regulation of inflammatory response | GO:0050728 | 0.000298 | 3.525115 | 151 | 31 | 3 |
| tube morphogenesis | GO:0035239 | 0.000309 | 3.510664 | 854 | 31 | 5 |
| cell migration | GO:0016477 | 0.000369 | 3.432481 | 1461 | 31 | 6 |
| regulation of cell migration | GO:0030334 | 0.000369 | 3.432466 | 893 | 31 | 5 |
| regulation of cell motility | GO:2000145 | 0.000486 | 3.313061 | 952 | 31 | 5 |
| response to reactive oxygen species | GO:0000302 | 0.000569 | 3.244973 | 193 | 31 | 3 |
| positive regulation of cell migration | GO:0030335 | 0.000595 | 3.225698 | 523 | 31 | 4 |
| tube development | GO:0035295 | 0.000727 | 3.138695 | 1054 | 31 | 5 |
| negative regulation of cytokine production | GO:0001818 | 0.001441 | 2.841199 | 278 | 31 | 3 |
| cell morphogenesis involved in differentiation | GO:0000904 | 0.001747 | 2.75767 | 722 | 31 | 4 |
| leukocyte activation | GO:0045321 | 0.004168 | 2.380078 | 953 | 31 | 4 |
| regulation of cell population proliferation | GO:0042127 | 0.00429 | 2.367553 | 1658 | 31 | 5 |
| cellular nitrogen compound biosynthetic process | GO:0044271 | 0.005971 | 2.223989 | 4765 | 31 | 8 |
| positive regulation of apoptotic process | GO:0043065 | 0.006135 | 2.212171 | 505 | 31 | 3 |
| positive regulation of programmed cell death | GO:0043068 | 0.006522 | 2.185648 | 518 | 31 | 3 |
| angiogenesis | GO:0001525 | 0.006588 | 2.181237 | 521 | 31 | 3 |
| regulation of lymphocyte activation | GO:0051249 | 0.006868 | 2.163194 | 529 | 31 | 3 |
| autophagy | GO:0006914 | 0.00713 | 2.146897 | 541 | 31 | 3 |
| chemotaxis | GO:0006935 | 0.009409 | 2.026437 | 613 | 31 | 3 |
| regulation of leukocyte activation | GO:0002694 | 0.009686 | 2.013859 | 620 | 31 | 3 |
| negative regulation of cell differentiation | GO:0045596 | 0.011272 | 1.948011 | 660 | 31 | 3 |
| lymphocyte activation | GO:0046649 | 0.016359 | 1.786249 | 794 | 31 | 3 |
| cellular response to cytokine stimulus | GO:0071345 | 0.018012 | 1.744437 | 831 | 31 | 3 |
| positive regulation of cell population proliferation | GO:0008284 | 0.022396 | 1.649835 | 929 | 31 | 3 |
