# Supplementary material for: Diabetic microenvironment deteriorates the regenerative capacities of adipose mesenchymal stromal cells
Source: Diabetol Metab Syndr. 2024 Jun 16;16:131. doi: 10.1186/s13098-024-01365-1 (PMC11181634; doi:10.1186/s13098-024-01365-1)

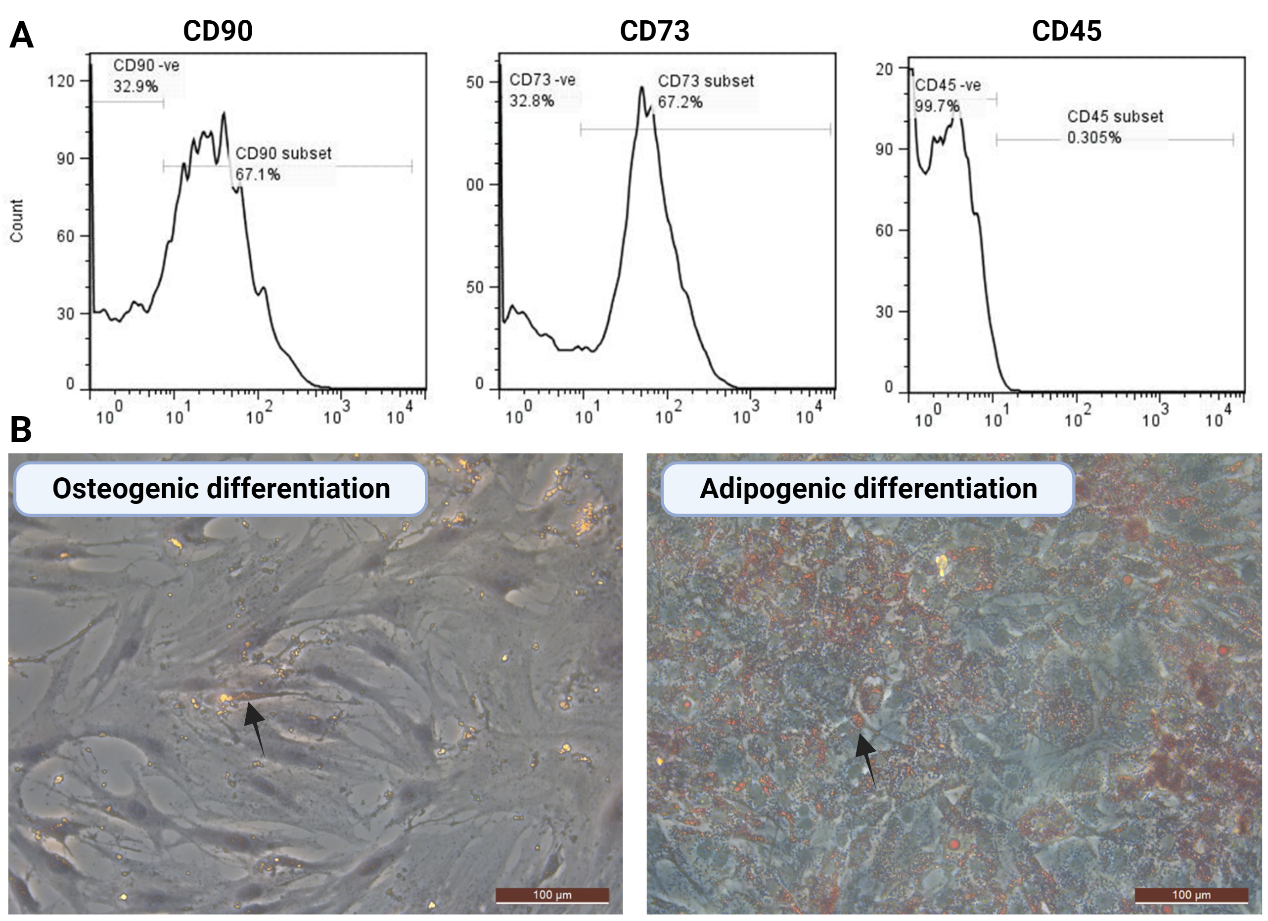


Supplementary figure 1 A-B: A) phenotypic characterization of ASCs for the MSC marker; CD90 and CD73 and the hematopoietic marker, CD45. ASCs is positive for CD90 and CD73, but negative for CD45. B) Osteogenic and adipogenic differentiation of ASCs. ASCs showed oil red o staining (arrow) denoting their adipogenic differentiation and Alizarin red stain (arrow) denoting osteogenic differentiation.


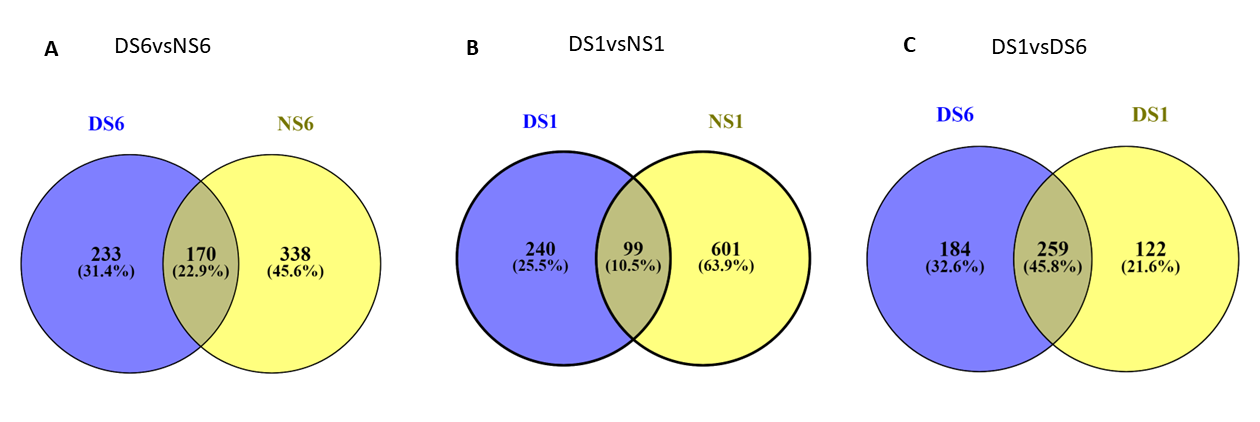


Supplementary figure 2: Venn diagram showing the overlap between groups at different point of collection. NS refers to ASCs grown in normal serum while DS refers to ASCs grown in diabetic serum for 6 days and one month.


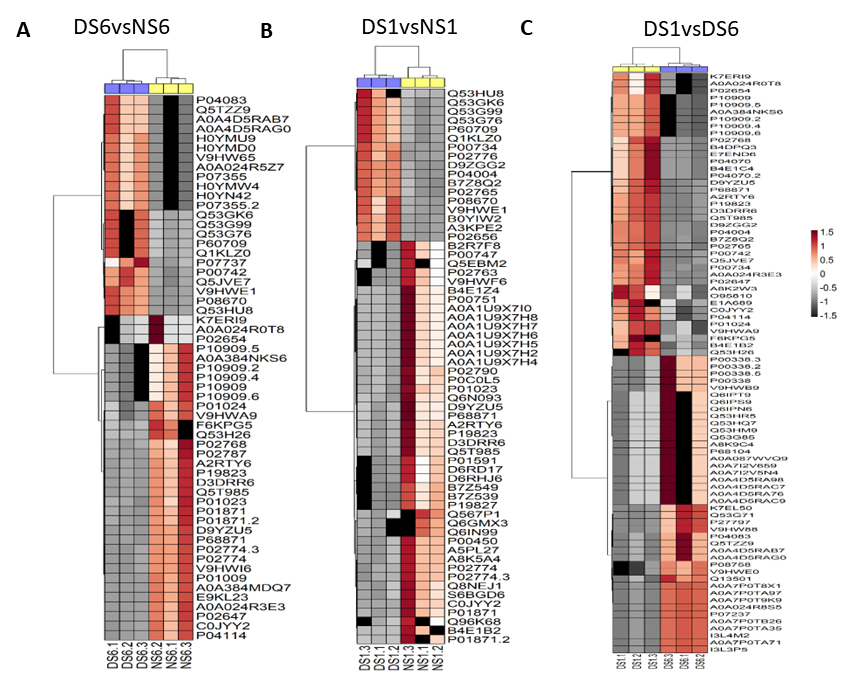


Supplementary figure 3: Heatmap FC2: Heatmap with cluster analysis using the Ward algorithm and the significant proteins from FC analysis (with less than two NAs values in each group). The legend shows the group color key.

Supplementary figure 4: ASCs were cultured in medium supplemented with diabetic serum (DS) (4A) and normal serum (NS) (4B) for 1, 2, 6 and 30 days**.** Longer culture caused morphological changes in ASCs as they became more flattened, acquired a granular texture, vacuolated cytoplasm (arrows).
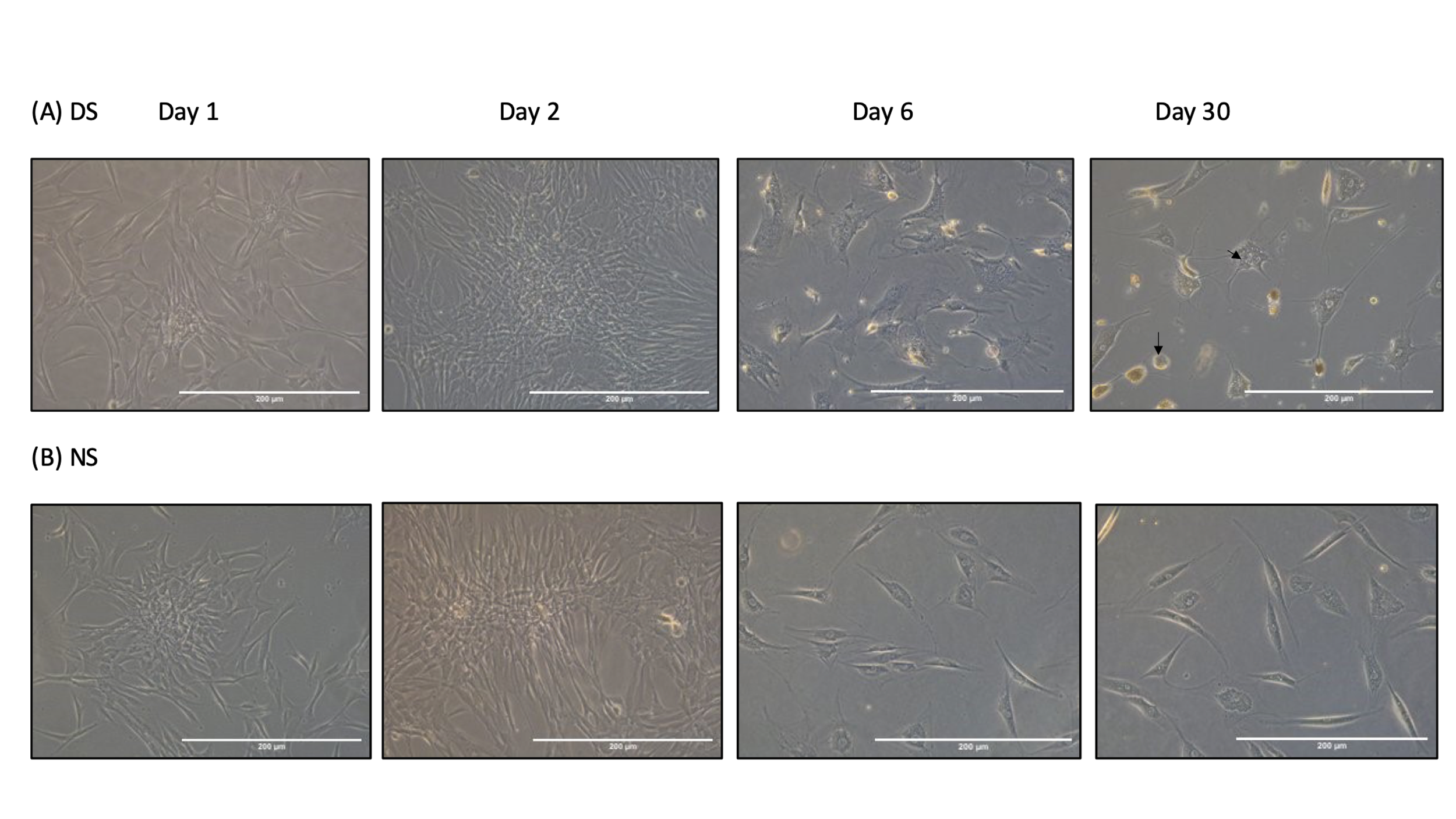

Supplement: Supplementary file 10 — Supplementary Material 10 [file 13098_2024_1365_MOESM10_ESM.docx]
