# Supplementary material for: Diabetic microenvironment deteriorates the regenerative capacities of adipose mesenchymal stromal cells
Source: Diabetol Metab Syndr. 2024 Jun 16;16:131. doi: 10.1186/s13098-024-01365-1 (PMC11181634; doi:10.1186/s13098-024-01365-1)
Supplement: Supplementary file 11 — Supplementary Material 11 [file 13098_2024_1365_MOESM11_ESM.docx]

| **Gene** | **Forward (5′→3′)** | **Reverse (5′→3′)** |
| --- | --- | --- |
| **Beta Actin** | **AGAGCTACGAGCTGCCTGC** | **AGCACTGTGTTGGCGTACAG** |
| **IGF1** | **GGCATAGCTGGCCAAACAA** | **CACTTGGGAGAAGGCTTAGAATAAA** |
| **IL-6** | **GTAGCCGCCCCACACAGAC** | **GCCATCTTTGGAAGGTTC** |
| **IL-8** | **GAACTGAGAGTGATTGAGT** | **CTTCTCCACAACCCTCTG** |
| **VEGFA** | **GGGCAGAATCATCACGAAT** | **ATCTGCATGGTGATGTTGGA** |
| **SIRT1** | **CGCTGGCCGACAACTTGTA** | **CATGTGAGGCTCTATCCTCCT** |
| **XRCC5** | **GACGTGGGCTTTACCATGAT** | **TCAGTGCCATCTGTACCAAAC** |
| **TNF-*α*** | **TCTTCTCGAACCCCGAGTGA** | **CCTCTGATGGCACCACCAG** |
| **CDKN1A** | **CCTGTCACTGTCTTGTACCCT** | **GCGTTTGGAGTGGTAGAAATCT** |
| **TERF1** | **AATGAAGGCAGCGGCAAAAG** | **AGTTACCGCAGACTGTTTGTC** |
| **TGF-*β*** | **CAGCAACAATTCCTGGCGATA** | **AAGGCGAAAGCCCTCAATTT** |
| **TSG-6** | **CATCTCGCAACTTACAAGC** | **AGACGGATTCCATAATCAATAATG** |
| **CXCR4** | **GGTGGTCTATGTTGGCGTCT** | **TGGAGTGTGACAGCTTGGAG** |
| **OCT4** | **TGTACTCCTCGGTCCCTTTC** | **TCCAGGTTTTCTTTCCCTAGC** |
| **SOX2** | **GCTAGTCTCCAAGCGACGAA** | **GCAAGAAGCCTCTCCTTGAA** |
| **Nanog** | **CAGTCTGGACACTGGCTGAA** | **CTCGCTGATTAGGCTCCAAC** |
| **PPARG** | **CTATGGAGTTCATGCTTGT** | **GTACTGACATTTATTT** |
| **RUNX2** | **AAGGGTCCACTCTGGCTTTG** | **CTAGGCGCATTTCAGGTGCT** |
| **COL1** | **CGGCTCCTGCTCCTCTTAG** | **CACACGTCTCGGTCATGGTA** |
| **IL-4R** | **CCTTGGGAAATCGATGAGAA** | **ACTGAACACCCCTTGACAGC** |
| **TNF-R1** | **CAGGAAGAACCAGTACCG** | **TTCTTACAGTTACTACAGGAGC** |
| **INFƴR** | **GGCAGCATCGCTTTAAACTC** | **GGAGGTGGGGGCTTTTATTA** |
| **IL-6R** | **GCAGGACCTCAGGTGAGAAG** | **TGGGAGGTGGAGAAGAGAGA** |
